# Optimizing infant HIV diagnosis with additional screening at immunization clinics in three sub‐Saharan African settings: a cost‐effectiveness analysis

**DOI:** 10.1002/jia2.25651

**Published:** 2021-01-20

**Authors:** Lorna Dunning, Aditya R Gandhi, Martina Penazzato, Djøra I Soeteman, Paul Revill, Simone Frank, Andrew Phillips, Caitlin Dugdale, Elaine Abrams, Milton C Weinstein, Marie‐Louise Newell, Intira J Collins, Meg Doherty, Lara Vojnov, Patricia Fassinou Ekouévi, Landon Myer, Angela Mushavi, Kenneth A Freedberg, Andrea L Ciaranello

**Affiliations:** ^1^ Medical Practice Evaluation Center Massachusetts General Hospital Boston MA USA; ^2^ Global HIV, Hepatitis, and STIs Programme World Health Organization Geneva Switzerland; ^3^ Center for Health Decision Science Harvard T.H. Chan School of Public Health Boston MA USA; ^4^ Center for Health Economics University of York York United Kingdom; ^5^ Institute for Global Health University College London London United Kingdom; ^6^ Division of Infectious Diseases Massachusetts General Hospital Boston MA USA; ^7^ Harvard Medical School Boston MA USA; ^8^ Mailman School of Public Health ICAP at Columbia University New York City NY USA; ^9^ Institute for Development Studies Human Development and Health Faculty of Medicine University of Southampton Southampton United Kingdom; ^10^ School of Public Health Faculty of Health Sciences University of Witwatersrand Johannesburg South Africa; ^11^ Medical Research Council Clinical Trials Unit University College London London United Kingdom; ^12^ Elizabeth Glaser Pediatric AIDS Foundation Abidjan Côte d’Ivoire; ^13^ Division of Epidemiology & Biostatistics School of Public Health & Family Medicine University of Cape Town Cape Town South Africa; ^14^ Ministry of Health and Child Care Harare Zimbabwe

**Keywords:** Early infant diagnosis, HIV, paediatric HIV testing, HIV‐exposed infants, immunization, prevention of mother‐to‐child HIV transmission

## Abstract

**Introduction:**

Uptake of early infant HIV diagnosis (EID) varies widely across sub‐Saharan African settings. We evaluated the potential clinical impact and cost‐effectiveness of universal maternal HIV screening at infant immunization visits, with referral to EID and maternal antiretroviral therapy (ART) initiation.

**Methods:**

Using the CEPAC‐Pediatric model, we compared two strategies for infants born in 2017 in Côte d’Ivoire (CI), South Africa (SA), and Zimbabwe: (1) existing EID programmes offering six‐week nucleic acid testing (NAT) for infants with known HIV exposure (*EID*), and (2) *EID* plus universal maternal HIV screening at six‐week infant immunization visits, leading to referral for infant NAT and maternal ART initiation (*screen‐and‐test*). Model inputs included published Ivoirian/South African/Zimbabwean data: maternal HIV prevalence (4.8/30.8/16.1%), current uptake of EID (40/95/65%) and six‐week immunization attendance (99/74/94%). Referral rates for infant NAT and maternal ART initiation after *screen‐and‐test* were 80%. Costs included NAT ($24/infant), maternal screening ($10/mother–infant pair), ART ($5 to 31/month) and HIV care ($15 to 190/month). Model outcomes included mother‐to‐child transmission of HIV (MTCT) among HIV‐exposed infants, and life expectancy (LE) and mean lifetime per‐person costs for children with HIV (CWH) and all children born in 2017. We calculated incremental cost‐effectiveness ratios (ICERs) using discounted (3%/year) lifetime costs and LE for all children. We considered two cost‐effectiveness thresholds in each country: (1) the *per‐capita* GDP ($1720/6380/2150) per year‐of‐life saved (YLS), and (2) the CEPAC‐generated ICER of offering 2 versus 1 lifetime ART regimens (e.g. offering second‐line ART; $520/500/580/YLS).

**Results:**

With *EID*, projected six‐week MTCT was 9.3% (CI), 4.2% (SA) and 5.2% (Zimbabwe). *Screen‐and‐test* decreased total MTCT by 0.2% to 0.5%, improved LE by 2.0 to 3.5 years for CWH and 0.03 to 0.07 years for all children, and increased discounted costs by $17 to 22/child (all children). The ICER of *screen‐and‐test* compared to *EID* was $1340/YLS (CI), $650/YLS (SA) and $670/YLS (Zimbabwe), below the *per‐capita* GDP but above the ICER of 2 versus 1 lifetime ART regimens in all countries.

**Conclusions:**

Universal maternal HIV screening at immunization visits with referral to EID and maternal ART initiation may reduce MTCT, improve paediatric LE, and be of comparable value to current HIV‐related interventions in high maternal HIV prevalence settings like SA and Zimbabwe.

## 1 INTRODUCTION

In 2019, more than 1.2 million infants were born to women with HIV worldwide and 150,000 acquired HIV [1]. Prompt diagnosis and treatment are critical to the survival of infants with HIV; without treatment, >50% will die before two years of age [2]. Diagnosis of infant HIV requires a nucleic acid test (NAT) because passively transferred maternal anti‐HIV antibodies cannot be differentiated from those endogenously produced in children with HIV (CWH) up to 18 months of age [3]. NAT‐based early infant HIV diagnosis (EID) is recommended for all infants with known HIV exposure (i.e. born to HIV status‐aware mothers), with prompt initiation of antiretroviral therapy (ART) for CWH [3, 4]. However, only 60% of HIV‐exposed infants were tested globally by two months of age in 2019, with 68% in Eastern and Southern Africa but only 33% in Western and Central Africa [1]. Low EID uptake is due partly to lack of knowledge of maternal HIV status (thus infant exposure), and loss to follow‐up before NAT is undertaken among infants known to be HIV‐exposed.

To improve access to EID, pilot projects have demonstrated the feasibility and acceptability of maternal HIV screening at infant six‐week expanded programme on immunization (EPI) visits, where attendance is often >90% [5, 6, 7, 8, 9]. This practice can identify HIV‐exposed infants not engaged in existing EID programmes and mothers who need ART, simultaneously improving maternal health and reducing the risk of HIV transmission to breastfed, HIV‐uninfected children. However, of concerns are the costs of these programmes, which require screening large numbers of women and, in some settings, may have low yield in identifying HIV‐exposed infants not engaged in EID services. We used a validated computer simulation model to examine the clinical impact and cost‐effectiveness of adding routine maternal HIV screening at immunization visits, with referral to infant HIV testing at existing EID programmes in Côte d’Ivoire (CI), South Africa (SA) and Zimbabwe.

## 2 METHODS

### 2.1 Model overview

We used the Cost‐Effectiveness of Preventing AIDS Complications (CEPAC)‐Pediatric model, a validated Monte Carlo simulation model of paediatric HIV acquisition, disease progression, diagnosis and treatment [10, 11, 12, 13, 14]. Children were simulated from birth until death. In the model, children who are HIV‐exposed have a risk of intrauterine or intrapartum HIV acquisition dependent on maternal ART use during pregnancy (reflecting prevention of mother‐to‐child transmission [PMTCT] coverage) and CD4 count (reflecting disease stage). Children who are HIV‐exposed but uninfected face a monthly risk of postnatal HIV acquisition based on maternal ART use, infant antiretroviral prophylaxis and maternal disease stage (including acute infection during breastfeeding) until cessation of breastfeeding, and no risk thereafter. All simulated children face monthly risks of non‐HIV‐related mortality. CWH face additional risks of opportunistic infections (OIs), and OI‐ and HIV‐related mortality based on their CD4% (age <5 years) or CD4 count (age ≥5 years), retention of care and ART use. Details of HIV disease progression; ART regimens, monitoring and outcomes; and loss to follow‐up and return to care, are provided in the Appendix and at https://mpec.massgeneral.org/cepac‐model/.

### 2.2 Population and strategies

We simulated all infants born in CI, SA and Zimbabwe in 2017 (the most recent year for which complete data were available), including infants born to women with and without HIV. These countries represent variation in key characteristics of the HIV epidemic, including maternal HIV prevalence (low/high/medium), EID uptake (low/moderate/high), maternal ART coverage (low/high/high) and income level (low/low/middle). Country guidelines recommend EID at six weeks of age for most infants in CI and Zimbabwe, and at birth and 10 weeks of age in SA; both are consistent with World Health Organization recommendations. We modelled six‐week EID for all countries to permit us to isolate the most influential parameters (EPI uptake, screening costs and care costs).

We compared two strategies in each setting: (1) six‐week NAT for infants with known HIV exposure (*EID*), and (2) *EID* plus universal maternal rapid diagnostic testing (RDT) at six‐week infant immunization visits, with positive result leading to referral for infant NAT and maternal ART initiation (*screen‐and‐test*). In *screen‐and‐test*, HIV status‐aware mothers who did not attend an EID visit at six weeks with their infant, but presented to an EPI visit, could be referred back to an EID clinic for NAT. In both strategies, we simulated a confirmatory NAT algorithm before ART initiation. Children who develop an OI and are not in care experience a probability of presenting to care, undergoing HIV testing and linking to care. Women with HIV were not directly simulated in either strategy; rather, maternal characteristics were reflected in changes in infant HIV acquisition risk over time.

### 2.3 Model input parameters

We derived clinical data to inform cohort characteristics, MTCT risks, assay characteristics and treatment outcomes from published trials and cohort studies in sub‐Saharan Africa (Table 1, Sections I‐III) [15, 16, 17, 18, 19, 20, 21, 22, 23, 24, 25, 26, 27, 28, 29, 30, 31, 32, 33, 34, 35]. We used Ivoirian, South African and Zimbabwean programmatic data for three separate country‐specific base‐case analyses (Table 1, Section IV), and varied these parameters from sensitivity analyses. We used national estimates of maternal HIV prevalence during pregnancy (4.8%, 30.8%, 16.1%) and postpartum maternal HIV incidence (0.4/100 person‐years (PY), 2.9/100PY, 1.5/100 PY) [36, 37, 38, 39]. Maternal knowledge of HIV status during pregnancy (86%, 89%, 84%) reflected the product of antenatal care (ANC) attendance (91%, 94%, 93%) and HIV testing coverage during ANC or at delivery (95%, 95%, 90%) [36, 37, 38, 40]. Maternal ART coverage during both pregnancy and breastfeeding (70%, 95%, 95%) and uptake of existing EID programmes (40%, 95%, 65%) were from UNAIDS data [36]. In *screen‐and‐test*, the probability of maternal screening was the product of six‐week immunization coverage (99%, 74%, 94%), which was derived from UNICEF country‐specific data, and a 90% probability of offer and acceptance of testing [5, 6, 7, 8, 9, 41]. At EPI visits, newly diagnosed mothers and HIV status‐aware mothers who missed an EID visit at six weeks had a modelled 80% probability of successful referral to existing EID programmes [42]. We assumed that the probability of maternal linkage to HIV care and ART after the EID visit was equal to country‐specific maternal ART coverage. Once diagnosed through any mechanism, including detection after OI, infants had a 71% probability of linking to HIV care and ART in all countries [43, 44].

**Table 1:** Selected base‐case input parameters for the CEPAC‐Paediatric model analysis of *EID* and *screen‐and‐test*

Variable	Base‐case value				References
I. Clinical input parameters					
Male infants, %	48				[48]
Mothers with CD4 ≤ 350/µL before ART, %	49				[83]
Infant CD4% at infection, mean (SD)	45 (10)				[11]
IU/IP MTCT (one‐time risk in pregnancy/delivery, %)	Maternal CD4 ≤ 350/µL		Maternal CD4 > 350/µL		
On ART	0.93		0.93		[15, 16, 17, 18]
Not on ART	27		17		[19, 20, 21, 22, 23, 24]
PP MTCT (monthly risk during breastfeeding, %)					
On ART	0.19		0.19		[25, 26, 27, 28, 29]
Not on ART					
Exclusive breastfeeding	0.76		0.24		[19, 30, 31, 32]
Mixed or complementary breastfeeding	1.28		0.40		[19, 30, 31, 32]
II. Assay characteristics					
NAT sensitivity, specificity for infant HIV, %					
IU infection: all ages, %	100, 99.6				[79, 84, 85, 86]
IP/PP infection: month in which infection occurs, %	0, 99.6				[79, 84, 85, 86]
IP/PP infection: subsequent months, %	100, 99.6				[79, 84, 85, 86]
RDT sensitivity, specificity for maternal HIV, %	99.9, 100				[87, 88]
III. Art Outcomes	(first‐line ART)		(second‐line ART)		
ART efficacy: HIV RNA < 400c/mL at 24 weeks on ART, %					
Ages <5 years	91		75		[33, 34]
Ages ≥5 years	75		75		[35]
CD4 count increase, mean CD4%/month, range by month	0.7 to 2.2		0.4 to 1.9		[33, 34]
Monthly loss to follow‐up after ART initiation, %	0.2				[89, 90]
IV. Country‐specific clinical parameters	Côte d’Ivoire	South Africa		Zimbabwe	
Antenatal					
Maternal HIV prevalence, %	4.8	30.8		16.1	[36, 91]
Maternal knowledge of HIV status, %^a^	86	89		84	[36, 37, 38, 40]
Postnatal					
Maternal HIV incidence (/100PY)	0.4	2.9		1.5	[39]
Mean breastfeeding duration, months	12	12		18	[37, 40, 92]
Proportion of infants breastfed from birth, %	80	80		80	[93, 94]
Breastfeeding for first six months: exclusive, mixed, %	25, 55	55, 25		55, 25	[94, 95] Assumption
Maternal ART coverage in pregnancy/breastfeeding (PMTCT), %	70	95		95	[36]
Routine 6‐week EID for infants with known HIV exposure:					
Uptake of existing EID programmes, %	40	95		65	[36]
Linkage to care/ART after positive EID test, %	71	71		71	[43, 44]
Maternal HIV testing at infant immunization visits					
Immunization coverage (six to ten weeks), %	99	74		94	[41]
Offer and acceptance of maternal RDT, %	90	90		90	[5, 7, 8, 9]
Linkage to care/ART for newly diagnosed mothers, %	80	80		80	[42]
Linkage to NAT for HIV‐exposed infants, %	80	80		80	Assumption
Linkage to care/ART for diagnosed infants referred from EPI, %	71	71		71	[43, 44]
V. Costs (2018 USD)	Côte d’Ivoire	South Africa		Zimbabwe	
Routine HIV care, per month (range by CD4%/count)^c^	20 to 190	15 to 140		30 to 35	[54, 55, 56, 57, 58]
Acute OI care (range by type of OI)	60 to 480	210 to 1,490		–^b^	[54, 55, 56, 57, 58]
Paediatric ART, per month (range by ART regimen)	5 to 31	5 to 31		5 to 31	[59, 60]
NAT, per assay	24	24		24	[62]
Maternal screening programme, per mother–infant pair^d^	10	10		10	[61]

ANC, antenatal care; ART, antiretroviral therapy; EID, early infant diagnosis; EPI, expanded programme on immunization; IP, intrapartum; IU, intrauterine; MTCT, mother‐to‐child transmission; NAT, nucleic acid test; OI, opportunistic infection; PP, postpartum; PY, person‐years; RDT, rapid diagnostic test; SD, standard deviation.

^a^ Maternal knowledge of HIV status was calculated from the product of ANC coverage and frequency of HIV testing in ANC in each country.

^b^ Based on available data, for CI and SA we modelled costs of care for individual OIs; in Zimbabwe, OI care was included in overall monthly care costs.

^c^ CD4% is used for ages <5 years, CD4 count used for ages ≥5.

^d^ Overall cost reflects both the cost of a maternal rapid diagnostic test and programme implementation costs.

We derived country‐ and sex‐ specific mortality rates for HIV‐unexposed children from UNAIDS HIV‐deleted life tables, and mortality rates for HIV‐exposed/uninfected infants from pooled UNAIDS analyses [45, 46]. Therefore, life expectancy (LE) projections are not expected to be directly comparable across country settings. Risks of disease progression without ART were calibrated to survival data for African children and adults [11, 47, 48, 49, 50, 51, 52]. Survival and OI risks for children and adults on ART were calibrated to clinical trial data [11, 33, 34, 35, 49, 53].

We modelled costs of HIV testing and clinical care in 2018 USD (Table 1, Section V). Costs specific to each country included routine HIV care (e.g., laboratory monitoring, personnel, facilities) and acute OI care [54, 55, 56, 57, 58]. ART costs ranged by age and weight ($5 to 31/month) and were derived from Clinton Health Access Initiative price lists and World Health Organization weight‐based dosing [59, 60]. Assay costs were modelled as “fully loaded,” including personnel time and training, and were derived from Global Fund and published HIV testing reports: NAT ($24/assay), maternal HIV screening ($10/mother–infant pair: $3 for RDT plus personnel and training costs; Appendix p3) and ART monitoring (CD4: $5 to 12/assay; HIV RNA: $17 to 32/assay) [56, 61, 62, 63, 64]. In *screen‐and‐test*, per‐person lifetime costs included the cost of maternal ART during breastfeeding for mothers diagnosed and linked to care through screening.

### 2.4 Model outcomes

Primary model outcomes were MTCT proportion at six weeks and after weaning, incremental yield of the screening programme (the additional number of infants identified with HIV divided by the number of women reached by the *screen‐and‐test* programme), proportion of all CWH identified and linked to care, 1‐ and 2‐year survival, LE (years) and average per‐person lifetime costs from a healthcare system perspective (2018 USD). We projected outcomes for both CWH and the complete birth cohort (including CWH, uninfected children with HIV exposure and HIV‐unexposed children), but not for mothers.

Using discounted (3%/year) birth cohort outcomes, we calculated incremental cost‐effectiveness ratios (ICERs) in USD per year‐of‐life saved ($/YLS). In the absence of consensus about country‐specific cost‐effectiveness thresholds, we compared ICERs to (1) the 2018 *per‐capita* GDP in each country (CI: $1720/YLS, SA: $6380/YLS, Zimbabwe: $2150/YLS), and (2) the CEPAC‐generated ICER of a paediatric HIV programme offering 2 versus 1 lifetime ART regimens (e.g., offering second‐line ART; CI: $520/YLS, SA: $500/YLS, Zimbabwe: $580/YLS) [65, 66, 67, 68]. This ICER can be used to estimate the health benefits that would be foregone by diverting resources from an existing programme to a novel intervention, as a reasonable proxy for the value of alternative claims upon limited resources for HIV services. We varied cost‐effectiveness thresholds in sensitivity analyses.

### 2.5 Scenario analysis

We examined the impact of a birth and 10‐week EID schedule in SA. Modelled EID coverage was 67% at birth and 80% at 10 weeks [69]. HIV status‐aware mothers who presented to an EPI visit at six weeks, but whose infant did not receive a test at birth, could be referred back to an EID clinic in the next month.

### 2.6 Univariate and multivariate sensitivity analyses

We followed international guidance on uncertainty analysis and reported extensive univariate and multivariate uncertainty analyses, using literature‐based estimates of the uncertainty around key parameters [70]. For each country, we varied key epidemic‐specific parameters, uptake at each care “cascade” step for *EID* and *screen‐and‐test*, and costs of diagnostics and HIV care. We first varied these parameters through their published ranges, where available, to identify the impact of data uncertainty on results, including: maternal HIV prevalence, knowledge of HIV status, HIV incidence and ART coverage during pregnancy and breastfeeding; uptake of existing EID programmes; immunization coverage; and cost of infant NAT. We next evaluated wider ranges for remaining parameters where data‐informed ranges were unavailable (e.g. linkage to EID after screening and screening costs) in order to identify the threshold values at which *screen‐and‐test* would reach each cost‐effectiveness threshold. Table S1 shows the ranges through which parameters were varied. In multivariate sensitivity analyses, we varied the most influential individual parameters simultaneously.

## 3 RESULTS

### 3.1 Base‐case results: clinical outcomes

In CI (Table 2, top rows), 5.2% of infants was projected to have HIV exposure during pregnancy/breastfeeding. Among HIV‐exposed infants, projected six‐week MTCT was 9.3% in both strategies; total MTCT at weaning was 11.7% with *EID* and 11.5% with *screen‐and‐test*. Most infections occurred among infants born to mothers who were unaware of their status; in this group, six‐week MTCT was 21.9% in both strategies and total MTCT at weaning was 25.2% with *EID* and 23.8% with *screen‐and‐test*. Table 2 also shows corresponding results for SA and Zimbabwe. For all infants born in 2017, *screen‐and‐test* was projected to save 13 550 to 29 680 life‐years in CI, SA and Zimbabwe (Table S2). The incremental yield for the *screen‐and‐test* programme was 0.20% in CI, 0.53% in SA and 0.42% in Zimbabwe, suggesting that a programme would need to screen 500 mothers in CI, 190 in SA and 240 in Zimbabwe to identify one additional infant with HIV, compared to *EID* alone.

**Table 2:** Base‐case model projections of *EID* and s*creen‐and‐test* in Côte d’Ivoire, South Africa and Zimbabwe

		**MTCT outcomes** ^a^		**Life expectancy** ^b^			**Economic outcomes (birth cohort)** ^b^		
	HIV‐exposed	6‐week MTCT	18‐month MTCT	CWH (undiscounted)	Birth cohort (undiscounted)	Birth cohort (discounted)	Lifetime costs (undiscounted)	Lifetime costs (discounted)	ICER
	%	%	%	years	years	years	USD 2018	USD 2018	$/YLS
Côte d’Ivoire									
EID	5.2	9.3	11.7	20.42	65.72	26.86	80	40	
Screen‐and‐test	5.2	9.3	11.5	23.90	65.75	26.87	100	60	1340
South Africa									
EID	32.8	4.2	6.2	19.74	63.26	26.51	280	160	
Screen‐and‐test	32.8	4.2	6.0	21.69	63.33	26.54	310	180	650
Zimbabwe									
EID	17.3	5.2	8.5	19.77	64.58	26.42	140	80	
Screen‐and‐test	17.3	5.2	8.0	22.49	64.65	26.45	170	100	670
Scenario Analysis: EID at birth and 10 weeks in South Africa									
EID	32.8	4.2	6.2	19.77	63.26	26.51	280	160	
Screen‐and‐test	32.8	4.2	6.0	21.95	63.33	26.54	310	180	620

CWH, children with HIV; ICER, incremental cost‐effectiveness ratio; MTCT, mother‐to‐child transmission; USD, United States dollar; YLS, year‐of‐life saved.

^a^ MTCT outcomes are reported among all HIV‐exposed infants.

^b^ Life expectancies are rounded to two decimals, costs are rounded to the nearest $10. ICERs were calculated from discounted (3%/year) life expectancies and costs prior to rounding.

**Figure 1:**
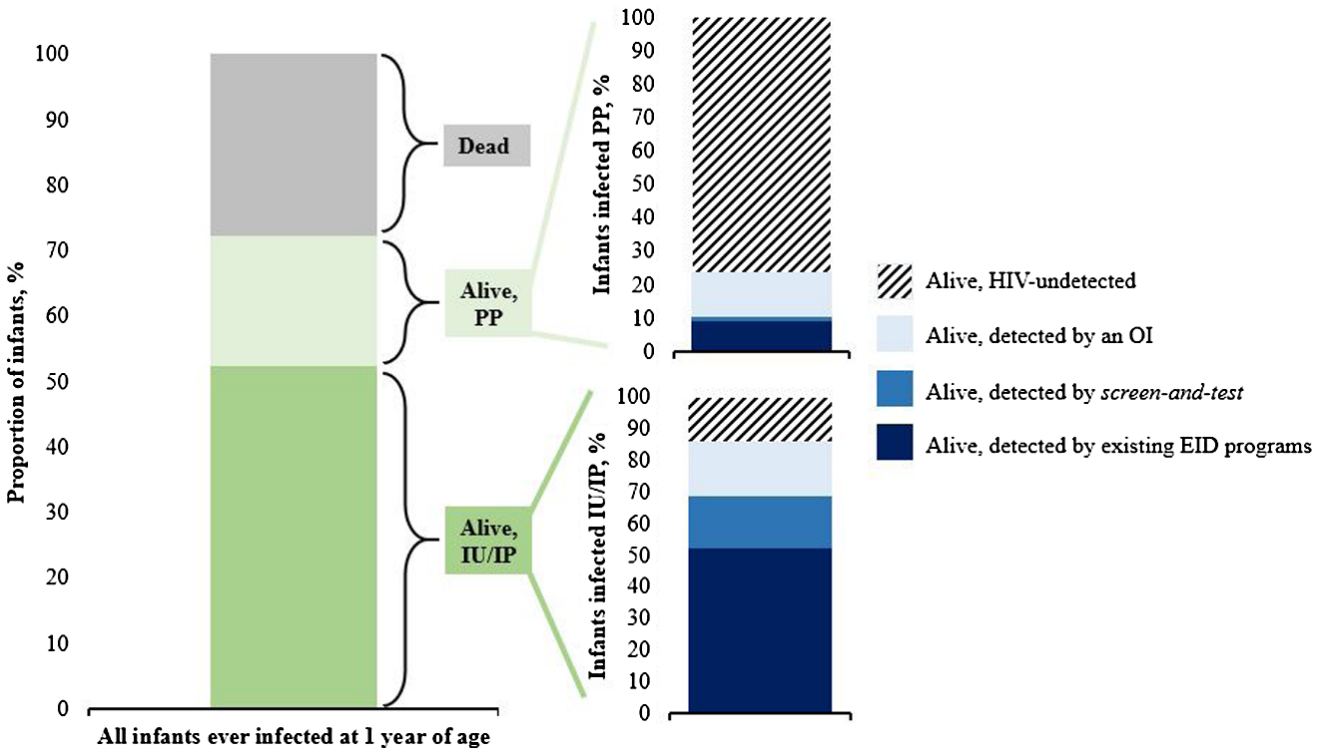
Mechanisms of HIV detection among children ever infected with HIV at 1 year from birth in the *screen‐and‐test* strategy in Côte d’Ivoire. Bar graph representing mechanisms of HIV detection among simulated infants with the proposed *screen‐and‐test* strategy. The left bar represents the proportions alive and dead at 1 year from birth of all infants who had acquired HIV by that time; results are reported separately for infants who acquired HIV during the IU/IP (dark green) vs. PP (light green) periods. The bottom right (IU/IP) and top right (PP) bars provide further details about the proportion of infants alive at 12 months of age who are undetected or were detected by an OI, existing EID programmes, or the *screen‐and‐test* programme. Similar results were observed in South Africa and Zimbabwe (see Appendix Table S3). Abbreviations: EID, early infant diagnosis; IP, intrapartum; IU, intrauterine; OI, opportunistic infection; PP, postpartum.

Among CWH, *EID* led to lower projected two‐year survival (CI: 58.5%, SA: 58.7%, Zimbabwe: 60.3%) compared to *screen‐and‐test* (CI: 67.3%, SA: 63.7%, Zimbabwe: 67.1%; Figure S1). *Screen‐and‐test* increased the proportion of infants with intrauterine or intrapartum HIV acquisition detected before development of an OI or death (Figure 1, Table S3). In CI, the LE for CWH was 20.42 years with *EID* and 23.90 years with *screen‐and‐test* (Table 2). Gains in LE followed a similar trend in SA (*EID:* 19.74 years, *screen‐and‐test:* 21.69 years) and Zimbabwe (*EID:* 19.77 years, *screen‐and‐test:* 22.49 years).

The impact of *EID* and *screen‐and‐test* on the survival of the entire birth cohort was smaller, because HIV‐unexposed infants, who made up the majority of the population, did not benefit from either strategy. For example, two‐year survival in CI was 94.21% with *EID* and 94.26% with *screen‐and‐test*, and LE was 65.72 and 65.75 years respectively (Table 2).

### 3.2 Base‐case results: lifetime costs and cost‐effectiveness

With *EID*, mean discounted per‐person lifetime HIV‐related costs for the entire birth cohort were $40 in CI, $160 in SA and $80 in Zimbabwe (Table 2). *Screen‐and‐test* increased projected costs by $20/child in all settings (to $60/infant in CI, $180/infant in SA and $100/infant in Zimbabwe), reflecting not only the additional cost of screening and EID programmes, but also greater costs for clinical care and ART as more infants were diagnosed and linked to HIV care and treatment (Figure S2).

Lifetime cost‐effectiveness results showed that the ICER of *screen‐and‐test* versus *EID* was $1340/YLS in CI (78% of the *per‐capita* GDP; 258% of the ICER of 2 versus 1 lifetime ART regimens), $650/YLS in SA (10% of the *per‐capita* GDP; 130% of the ICER of 2 versus 1 lifetime ART regimens) and $670/YLS in Zimbabwe (31% of the *per‐capita* GDP; 116% of the ICER of 2 versus. 1 lifetime ART regimens).

### 3.3 Scenario analysis

Compared to the base case, a birth and 10‐week EID schedule in SA resulted in slightly greater clinical benefit for CWH in both the *EID* and *screen‐and‐test* strategies (19.77 and 21.95 years respectively) due to more opportunities for EID (Table 2), but similar results for the entire birth cohort within rounding, and a similar ICER of *screen‐and‐test* versus *EID*.

### 3.4 Univariate sensitivity analyses

**Figure 2:**
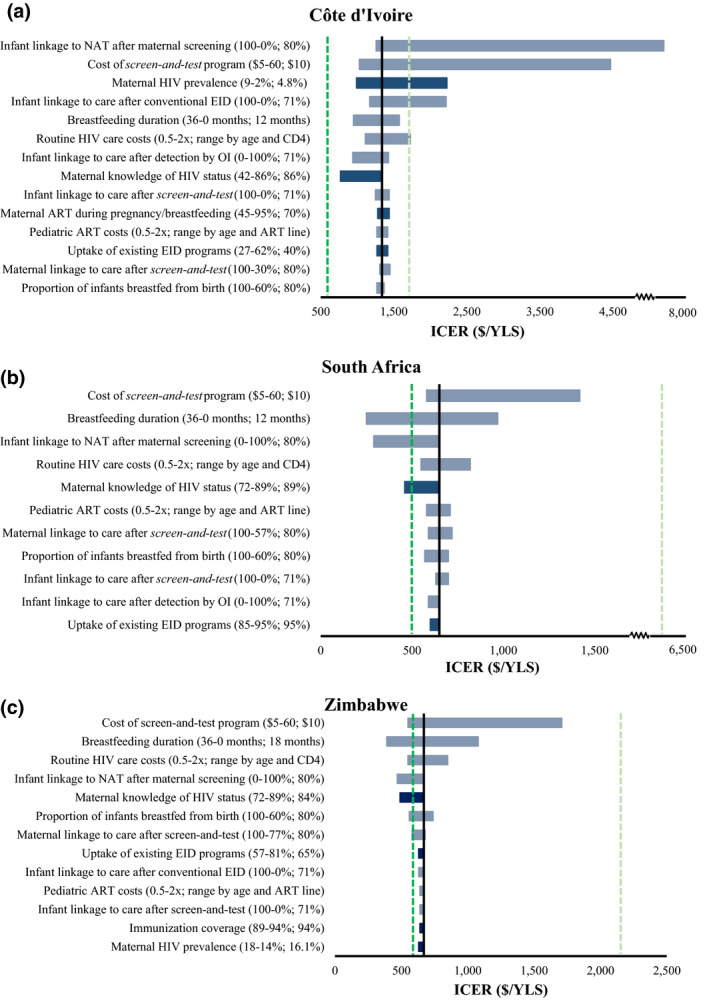
Univariate sensitivity analysis examining the impact of key input parameters on the cost‐effectiveness of *screen‐and‐test* compared to *EID* in (**A**) Côte d’Ivoire, (**B**) South Africa and (**C**) Zimbabwe. Univariate sensitivity analyses describing the impact of key input parameters on cost‐effectiveness results. The horizontal axis shows the incremental cost‐effectiveness ratio of *screen‐and‐test* compared to *EID*. The range through which each parameter is varied is shown in parentheses (value leading to the lowest shown ICER first, followed by value leading to the greatest shown ICER, with base‐case value after the semicolon). The length of each bar reflects the degree to which cost‐effectiveness is sensitive to variations in each parameter, with longest bars (greatest impact) at the top. Dark blue bars represent parameters for which published data ranges were available (data‐informed parameters, evaluated to understand the impact of parameter uncertainty on model outcomes); grey bars represent parameters for which no detailed data ranges were available (and thus wide ranges were evaluated to identify thresholds at which policy conclusions would change). The cost‐effectiveness criteria used are as follows: (1) the ICER of 2 versus 1 lifetime ART regimens (Côte d’Ivoire: $520/YLS; South Africa: $500/YLS; Zimbabwe: $580/YLS), and 2) the *per‐capita* GDP/YLS (Côte d’Ivoire: $1720/YLS; South Africa: $6380/YLS; Zimbabwe: $2150/YLS). Maternal HIV prevalence and incidence were varied together, holding the ratio of incidence to prevalence constant (0.008), to capture plausible variation in severity of the HIV epidemic. Several parameters did not influence the ICER of *screen‐and‐test* versus *EID* and thus are not shown here: In Côte d’Ivoire, the ICER of *screen‐and‐test* compared to *EID* was not sensitive to 3 parameters varied through data‐informed ranges (maternal HIV incidence [when varied alone], immunization coverage and the cost of infant NAT) and 1 parameter varied through wide ranges (acute OI care costs). In South Africa, the ICER of *screen‐and‐test* compared to *EID* was not sensitive to five parameters varied through data‐informed ranges (maternal HIV prevalence, maternal HIV incidence [when varied alone], immunization coverage, maternal ART coverage during pregnancy/breastfeeding and the cost of infant NAT) and 2 parameters varied through wide ranges (infant linkage to care after *EID* and acute OI care costs). In Zimbabwe, the ICER of *screen‐and‐test* compared to *EID* was not sensitive to 3 parameters varied through data‐informed ranges (maternal HIV incidence [when varied alone], maternal ART coverage during pregnancy/breastfeeding and the cost of infant NAT) and 1 parameter varied through wide ranges (infant linkage to care after detection by OI). All other input parameters shown in Table 1 were not influential on the ICER of *screen‐and‐test* versus *EID* in any country setting. Abbreviations: ART, antiretroviral therapy; EID, early infant diagnosis; ICER, incremental cost‐effectiveness ratio; NAT, nucleic test; YLS, year‐of‐life saved

Among parameters with available data‐informed ranges (Figure 2, blue bars), maternal HIV prevalence exerted the greatest influence on the cost‐effectiveness of *screen‐and‐test* versus *EID* in CI; with the lowest published prevalence (2%), the ICER exceeded the *per‐capita* GDP (Figure 2A). With higher prevalence or lower knowledge of maternal HIV status, as might be seen in sub‐national Ivoirian settings, the ICER was lower than in the base case, although never reached the ICER of 2 versus 1 lifetime ART regimens. In SA and Zimbabwe, maternal knowledge of HIV status had the greatest impact on the ICER of *screen‐and‐test* versus *EID* and the lowest reported value of maternal knowledge of HIV status led the ICER to fall below the ICER of 2 versus 1 lifetime ART regimens (Figures 2B,C).

Among parameters without available data‐informed ranges (Figure 2, grey bars), the ICER of *screen‐and‐test* versus *EID* was most sensitive to assumptions about screening programme costs, infant linkage to NAT after maternal screening, breastfeeding duration and routine HIV care costs in all three countries. In CI, even when the most favourable values for *screen‐and‐test* were assumed, the ICER never fell below $520/YLS (the ICER of 2 versus 1 lifetime ART regimens), although it was often below the *per‐capita* GDP (Figure 2A). In SA and Zimbabwe, the ICER was often near or below the ICER of 2 versus 1 lifetime ART regimens (falling below this threshold when infant linkage to NAT after maternal screening was low or breastfeeding duration was high), and never exceeded the *per‐capita* GDP (Figures 2B,C). Wide variations in all other key model input parameters did not substantially affect the ICER of *screen‐and‐test* versus *EID*.

### 3.5 Multivariate sensitivity analyses

**Figure 3:**
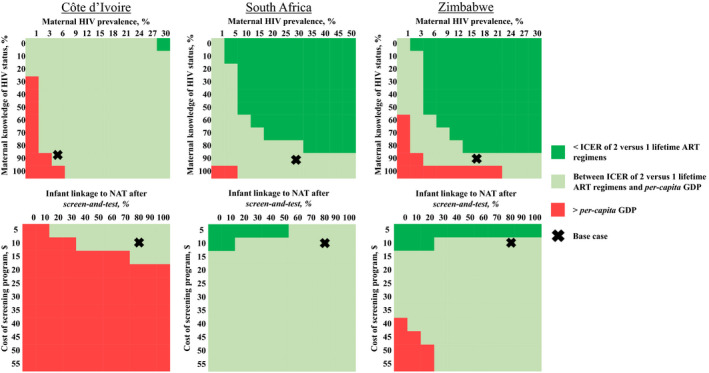
Multivariate analyses examining the impact of simultaneously varying maternal HIV prevalence and maternal knowledge of HIV status (top three panels), and infant linkage to NAT after maternal screening and the cost of the screening programme (bottom three panels) in Côte d’Ivoire, South Africa and Zimbabwe. Multivariate sensitivity analyses describing the joint impacts of maternal HIV prevalence and maternal awareness of HIV status (top three panels), and infant linkage to NAT after *screen‐and‐test* and the cost of the screening programme (bottom three panels) on cost‐effectiveness results. The cost‐effectiveness criteria used are as follows: (1) the ICER of 2 versus 1 lifetime ART regimens (Côte d’Ivoire: $520/YLS; South Africa: $500/YLS; Zimbabwe: $580/YLS), and 2) the *per‐capita* GDP/YLS (Côte d’Ivoire: $1720/YLS; South Africa: $6380/YLS; Zimbabwe: $2150/YLS). Red portions of the figure represent conditions where *screen‐and‐test* is not cost‐effective by either cost‐effectiveness criteria (the ICER of *screen‐and‐test* compared to *EID* is greater than the ICER of 2 versus 1 lifetime ART regimens and greater than the *per‐capita* GDP/YLS). Light green shading represents an ICER greater than the ICER of 2 versus 1 lifetime ART regimens but less than the *per‐capita* GDP/YLS. Dark green shading represents an ICER less than the ICER of 2 versus 1 lifetime ART regimens and less than the *per‐capita* GDP/YLS. Abbreviations: ART, antiretroviral therapy; ICER, incremental cost‐effectiveness ratio; NAT, nucleic acid test.

Figure 3 shows the joint impact of variation in key pairs of parameters: maternal HIV prevalence and awareness of HIV status (top three panels), and infant linkage to NAT after maternal screening and the cost of the screening programme (bottom three panels). In CI, plausible variations in maternal HIV prevalence or knowledge of HIV status led *screen‐and‐test* to meet the *per‐capita* GDP cost‐effectiveness threshold (light green shading) but not the ICER of 2 versus 1 lifetime ART regimens (dark green shading) (top left panel) [36, 71]. In CI, the *screen‐and‐test* programme (exclusive of subsequent NAT and ART) would need to achieve high (>50%) infant linkage to NAT and cost <$15/mother–infant pair to meet even the *per‐capita* GDP cost‐effectiveness threshold (bottom left panel).

The cost‐effectiveness of *screen‐and‐test* versus *EID* in SA and Zimbabwe followed similar directional trends, although with more scenarios in which *screen‐and‐test* was cost‐effective. *Screen‐and‐test* met one or both cost‐effectiveness thresholds except when maternal HIV prevalence was lower than the base case (SA: <10%, Zimbabwe: <15%) and maternal knowledge of HIV status was simultaneously high (SA: >90%. Zimbabwe: >70%) (top middle and right panels). *Screen‐and‐test* met one or both cost‐effectiveness thresholds in SA over all ranges explored for infant linkage to NAT and screening costs (bottom middle panel), and in Zimbabwe when infant linkage to NAT was >30% and the screening programme cost was <$35 (bottom right panel). When screening costs were <$10 in SA and <$5 in Zimbabwe, *screen‐and‐test* would still be cost‐effective even if infants were not diagnosed or linked to care, and the only benefits were a reduction in postpartum MTCT resulting from maternal diagnosis and ART initiation. In CI, in contrast, where maternal HIV prevalence is lower, *screen‐and‐test* would need to afford greater clinical benefit than just a reduction in postpartum MTCT to be cost‐effective.

## 4 DISCUSSION

In this model‐based analysis, we evaluated the clinical impact and cost‐effectiveness of a maternal HIV screening programme at immunization visits in Côte d’Ivoire, South Africa and Zimbabwe, with subsequent referral to EID and maternal ART initiation. We observed three key findings.

First, *screen‐and‐test* substantially improved LE for CWH and reduced postpartum MTCT in all three countries. For CWH, projected two‐year survival improved by an absolute amount of 5.0% to 8.8% and LE increased by 2.0 to 3.5 years, compared to *EID*. Although these gains are substantial compared to other medical therapies, they are accrued only to a small proportion of children in the birth cohort. Nonetheless, they were large enough to lead to gains in LE at the entire population‐level (0.36 to 0.84 life‐months) similar to projected LE gains from many other currently funded interventions [72]. Among children with HIV exposure, 18‐month MTCT decreased by 0.2% to 0.5%. Infants born to HIV status‐unaware mothers, who experience the greatest risk of acquiring HIV, benefited greatly from a *screen‐and‐test* programme that could facilitate infant and maternal diagnosis and ART initiation. Infants born to HIV status‐aware mothers also benefited, because EID uptake is not perfect (40% to 95%), and *screen‐and‐test* provided an additional opportunity for these infants to be re‐referred to EID at well‐child immunization visits.

Second, *screen‐and‐test* was more costly than *EID*. Greater per‐person lifetime costs with *screen‐and‐test* were due primarily due to caring for CWH over their lifetimes (Figure S2). Although costlier, *screen‐and‐test* was below the *per‐capita* GDP threshold in all three countries. *Screen‐and‐test* was more economically favourable in SA and Zimbabwe than in CI partly because fewer mothers would need to be screened to identify one additional infant with HIV compared to *EID* alone (190 in SA, 240 in Zimbabwe, and 500 in CI). Country differences were also due to key epidemic factors. In SA and Zimbabwe, where maternal HIV prevalence is high, cost‐effectiveness was due to more timely identification and treatment of infants living with HIV and a reduction in postpartum MTCT. Although CI has a lower maternal HIV prevalence, simultaneously low rates of maternal HIV testing and PMTCT uptake led to high numbers of infants missed by routine EID and contributed to the value of the *screen‐and‐test* strategy. In addition, in CI, because uptake of existing EID programmes is also low, the cost‐effectiveness was also due in part to a second opportunity for infant testing among HIV status‐aware mothers. The determination of whether health interventions are “cost‐effective” depends heavily upon a country’s willingness to pay for health. The widely cited *per‐capita* GDP‐based cost‐effectiveness threshold may be too high in resource‐limited settings: investing in health interventions with ICERs near the *per‐capita* GDP may forego health benefits by diverting resources from better‐value interventions [68, 73, 74, 75]. Although the ICER of *screen‐and‐test* versus *EID* was lower than the *per‐capita* GDP in all three countries, it exceeded the ICER of the benchmark intervention of offering 2 versus 1 lifetime ART regimens to CWH. However, the ICER of *screen‐and‐test* versus *EID* compares favourably to other funded HIV‐related interventions in SA and Zimbabwe, such as existing EID programmes compared to no EID (CEPAC‐generated ICERs of $1250/YLS and $1050/YLS respectively) [13, 14]. In low maternal HIV prevalence settings such as CI, *screen‐and‐test* would be a less valuable strategy than alternative investments such as paediatric care offering two ART regimens, but sensitivity analyses suggested potential cost‐effectiveness in subnational Ivoirian settings where maternal knowledge of HIV status is low or maternal HIV prevalence or breastfeeding duration is high.

Third, several key factors influenced the cost‐effectiveness of the *screen‐and‐test* versus *EID*. In all three countries, cost‐effectiveness depended on screening programme cost, infant linkage to NAT after referral from the screening programme, and maternal knowledge of HIV status during pregnancy. These findings have important implications for the potential implementation of *screen‐and‐test* programmes. There are minimal data to inform the proportion of HIV‐exposed infants that would link to NAT and HIV care if *screen‐and‐test* were implemented nationally. One recent study of intra‐facility linkage to HIV chronic care for mothers identified as HIV‐infected during ANC in Uganda found that only 37% of women transferred to a new clinic setting, and only 30% of all HIV‐exposed infants linked to EID programmes [76]. Even if linkage to infant NAT were this low, *screen‐and‐test* would still be cost‐effective in SA and Zimbabwe (Figure 3).

Although *screen‐and‐test* would improve clinical outcomes and likely be cost‐effective in SA and Zimbabwe, and perhaps in specific settings within CI, key questions about the implementation and relative value of alternative paediatric HIV case‐finding approaches remain unanswered. *Screen‐and‐test* would require screening large numbers of women in order to identify one CWH. Additional strategies to identify HIV‐exposed infants (e.g. maternal testing during pregnancy, labour and/or breastfeeding) and diagnose CWH (e.g. routine testing at nutrition, inpatient and tuberculosis clinics) may have different testing yields, clinical benefit and cost‐effectiveness. There is also limited knowledge about the feasibility of adding HIV testing into already busy EPI clinics, or about the amount of additional healthcare personnel time and training required. Additionally, a pilot effort to introduce HIV testing in Tanzanian EPI clinics reportedly reduced vaccine acceptance, perhaps due to concerns about HIV testing [9], although other pilot studies showed no such reduction [5, 6]. Lower vaccination rates and increased rates of vaccine‐preventable illness among children might outweigh the benefits of additional infant HIV diagnosis and prevention of postnatal HIV acquisition.

There are several limitations in this analysis. First, treatment availability, clinical care and healthcare costs are likely to change over infants’ lifetimes, rendering long‐term model‐based projections for children uncertain. We addressed this uncertainty by calibrating our model to currently available survival, MTCT risk and OI data, and varying epidemic‐specific and treatment parameters that are likely to change over time. Except where noted, model‐based policy conclusions were robust to plausible changes in these parameters. Second, we did not evaluate clinical outcomes, costs or potential reduced MTCT in subsequent pregnancies among mothers. The long‐term benefits and costs among newly diagnosed mothers would be expected to be of comparable or greater value to HIV testing programmes in adults, and including maternal outcomes would likely improve the cost‐effectiveness of *screen‐and‐test* [61, 77]; integrated maternal and infant HIV services has been shown to be cost‐effective for mother–infant pairs in SA [78]. Third, we modelled costs from a healthcare system perspective, which does not account for societal costs incurred (e.g. patient travel or lost work) or offset (e.g. productivity savings from averting paediatric HIV infections) as a result of a *screen‐and‐test* strategy. Lastly, we did not simulate alternative approaches to *screen‐and‐test*. For example, on‐site collection of infant heel‐stick samples in EPI clinics rather than referral to off‐site EID programmes (using either dried blood spots with shipment to central laboratories or point‐of‐care NATs onsite) would likely further improve the clinical benefits of *screen‐and‐test* programmes [5, 79, 80].

## 5 CONCLUSIONS

We found that screening for infant HIV exposure at the first infant immunization visit, followed by NAT for infants identified as exposed, would decrease MTCT among infants whose mothers are undiagnosed or not virologically suppressed on ART, would improve infant life expectancy among infants with HIV, and may be cost‐effective in South Africa and Zimbabwe. In a low maternal HIV prevalence setting like Côte d’Ivoire, *screen‐and‐test* is less likely to be cost‐effective relative to existing health interventions. Results were robust across a wide range of sensitivity analyses, indicating potential generalizability to a variety of high maternal HIV prevalence settings in sub‐Saharan Africa. Linkage to infant NAT, paediatric care and maternal care greatly influenced the projected infant life expectancy with both strategies, as well as the cost‐effectiveness of *screen‐and‐test,* and thus are critical components to averting infant HIV‐related mortality, reducing MTCT and ensuring the cost‐effectiveness of a *screen‐and‐test* strategy.

## ETHICS

This study was approved by the Partners Human Research Committee.

## DATA AND CONSENT

No patient‐level data were included in this modelling study; only published data were included, so no consent was required.

## COMPETING INTEREST

The authors have no conflicts of interest.

## AUTHORS’ CONTRIBUTIONS

Designed the analysis: LD, AG, MP, AC. Designed and implemented revisions to the CEPAC model: LD, CD, MCW, KAF, AC. Conducted the analysis: LD, AG. Reviewed model results and provided critical interpretation: LD, AG, MP, DIS, PR, SF, AP, CD, EA, MCW, MN, IJC, MD, LV, PFE, LM, AM, KAF, ALC. Drafted the manuscript: LD, AG, AC. Critically revised the manuscript: LD, AG, MP, DIS, PR, SF, AP, CD, EA, MCW, MN, IJC, MD, LV, PFE, LM, AM, KAF, ALC. Read and approved the final manuscript: LD, AG, MP, DIS, PR, SF, AP, CD, EA, MCW, MN, IJC, MD, LV, PFE, LM, AM, KAF, ALC.

## Supporting information


**Figure S1.** Two‐year survival of infants with HIV diagnosed by *EID* only and with addition of *screen‐and‐test* in Côte d’Ivoire (top left panel), South Africa (top right panel), and Zimbabwe (bottom left panel).

**Figure S2.** Total lifetime costs per infant by HIV testing strategy in Côte d’Ivoire, South Africa, and Zimbabwe.

**Table S1.** Select base case data parameters and ranges for the CEPAC‐Pediatric model analysis of *EID* and *screen‐and‐test*
**Table S2.** Discounted life expectancy for the birth cohort in Côte d’Ivoire, South Africa, and Zimbabwe
**Table S3.** Outcomes and mechanisms of HIV detection among children ever infected with HIV at 1 year from birth in the *screen‐and‐test* strategy in Côte d’Ivoire, South Africa, and Zimbabwe
**Data S1.** Online decision support tool: CEPAC model outputs from this analysis are the basis for an online webtool (https://www.who.int/publications‐detail/paediatric‐hiv‐testing‐strategy‐decision‐tool) designed to optimize testing strategies at national and subnational levels, to promote future approaches tailored to specific epidemic and programmatic contexts [81, 82].
