# Supplementary figures and images for: Optimizing infant HIV diagnosis with additional screening at immunization clinics in three sub‐Saharan African settings: a cost‐effectiveness analysis
Source: J Int AIDS Soc. 2021 Jan 20;24(1):e25651. doi: 10.1002/jia2.25651 (PMC8992471; doi:10.1002/jia2.25651)

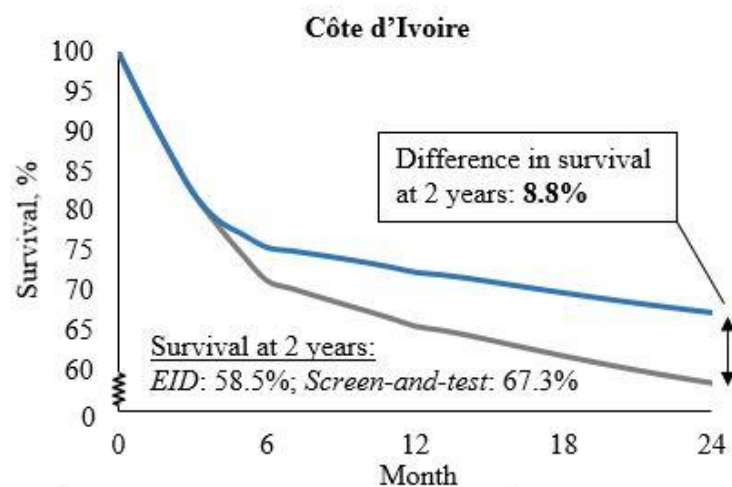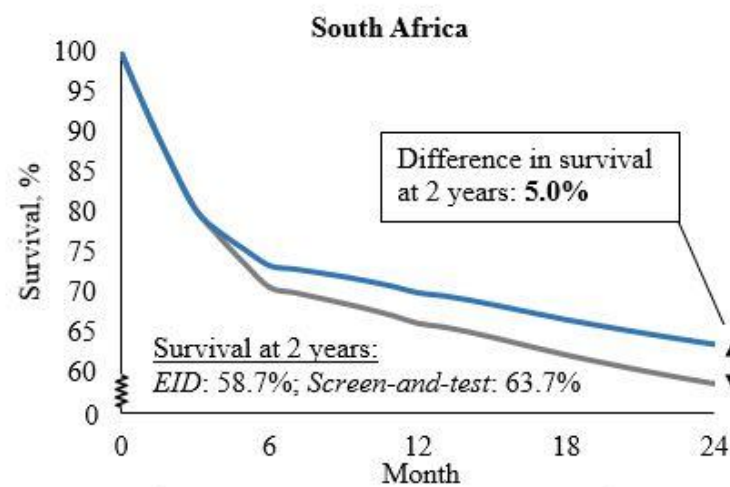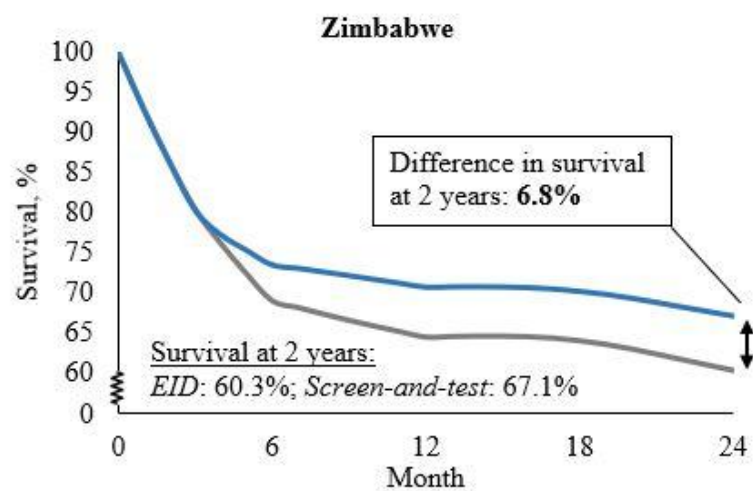

**Figure legend:**

— EID

— Screen-and-test

Supplement: Supplementary file 1 — Figure S1. Two‐year survival of infants with HIV diagnosed by EID only and with addition of screen‐and‐test in Côte d’Ivoire (top left panel), South Africa (top right panel), and Zimbabwe (bottom left panel). [file JIA2-24-e25651-s003.pdf]
