# Supplementary material for: Optimizing infant HIV diagnosis with additional screening at immunization clinics in three sub‐Saharan African settings: a cost‐effectiveness analysis
Source: J Int AIDS Soc. 2021 Jan 20;24(1):e25651. doi: 10.1002/jia2.25651 (PMC8992471; doi:10.1002/jia2.25651)
Supplement: Supplementary file 2 — Figure S2. Total lifetime costs per infant by HIV testing strategy in Côte d’Ivoire, South Africa, and Zimbabwe. [file JIA2-24-e25651-s002.pdf]

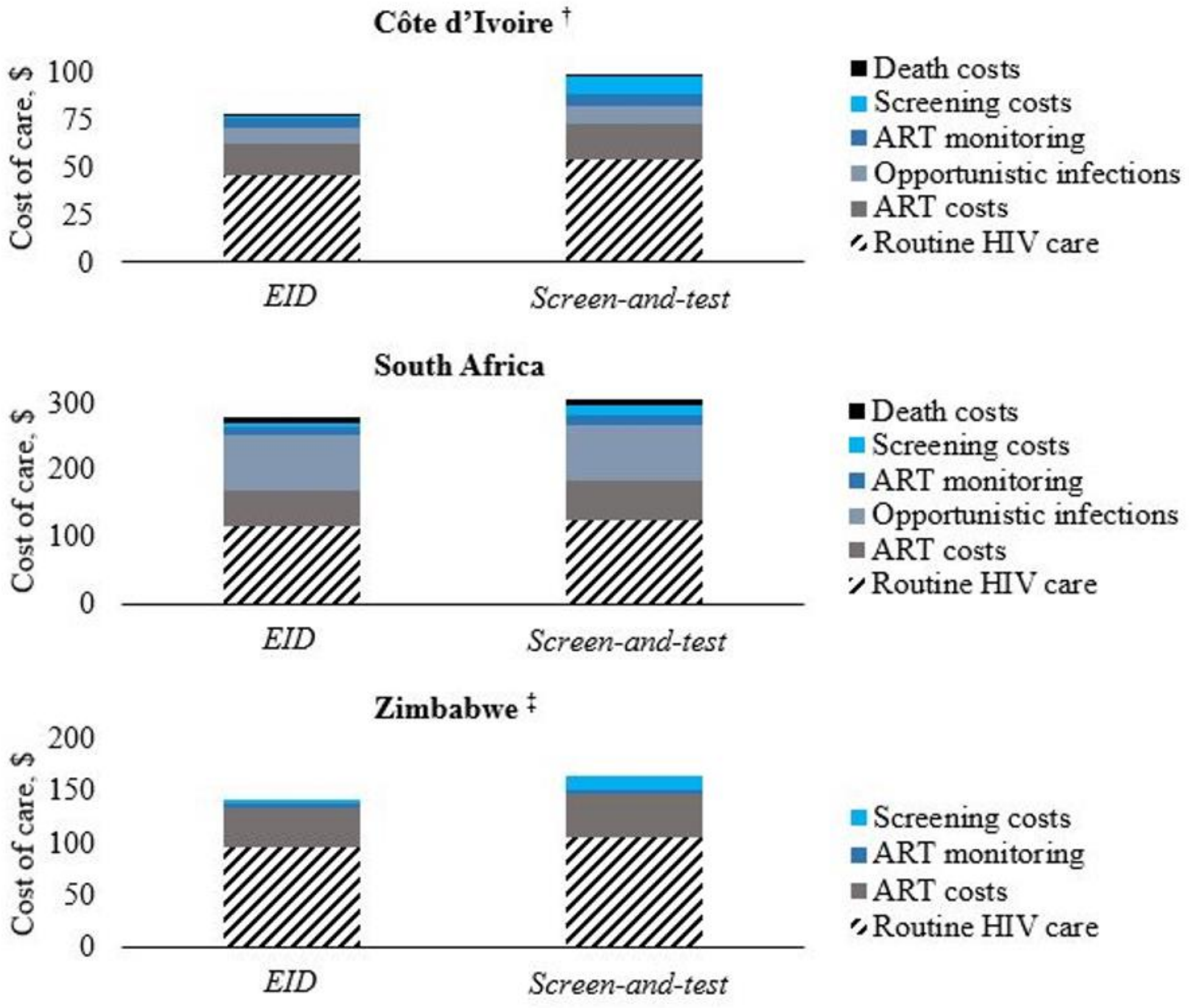

† Screening costs in *EID* and death costs in both strategies are <$1/infant.

‡ Opportunistic infection treatment costs and death costs in Zimbabwe are included in the cost of routine care.
