# Supplementary material for: Optimizing infant HIV diagnosis with additional screening at immunization clinics in three sub‐Saharan African settings: a cost‐effectiveness analysis
Source: J Int AIDS Soc. 2021 Jan 20;24(1):e25651. doi: 10.1002/jia2.25651 (PMC8992471; doi:10.1002/jia2.25651)
Supplement: Supplementary file 3 — Table S1. Select base case data parameters and ranges for the CEPAC‐Pediatric model analysis of EID and screen‐and‐test Table S2. Discounted life expectancy for the birth cohort in Côte d’Ivoire, South Africa, and Zimbabwe Table S3. Outcomes and mechanisms of HIV detection among children ever infected with HIV at 1 year from birth in the screen‐and‐test strategy in Côte d’Ivoire, South Africa, and Zimbabwe Data S1. Online decision support tool: CEPAC model outputs from this analysis are the basis for an online webtool (https://www.who.int/publications‐detail/paediatric‐hiv‐testing‐strategy‐decision‐tool) designed to optimize testing strategies at national and subnational levels, to promote future approaches tailored to specific epidemic and programmatic contexts [81, 82]. [file JIA2-24-e25651-s001.docx]

**Optimizing infant HIV diagnosis with additional screening at immunization clinics in three sub-Saharan African settings: A cost-effectiveness analysis**

**Supplementary Appendix**

Lorna Dunning, MBiochem, MPH^*^

Aditya R. Gandhi, BA^*^

Martina Penazzato, MD, MSc, PhD

Djøra I. Soeteman, PhD

Paul Revill, MSc

Simone Frank, BA

Andrew Phillips, PhD

Caitlin Dugdale, MD

Elaine Abrams, MD

Milton C. Weinstein, PhD

Marie-Louise Newell, MB, PhD

Intira J. Collins, PhD

Meg Doherty, MD

Lara Vojnov, PhD

Patricia Fassinou Ekouévi, MD

Landon Myer, MD, PhD

Angela Mushavi, MBChB, MMed

Kenneth A. Freedberg, MD, MSc
Andrea L. Ciaranello, MD, MPH

**INTRODUCTION**

This Appendix is included to provide methodologic details to supplement the description of the methods in the manuscript text, as well as additional model output and results. For reader convenience, this Appendix summarizes information about the Cost-Effectiveness of Preventing AIDS Complications (CEPAC)-Pediatric model structure that has also been described in the technical appendices of prior publications [1,2].

**METHODS**

**Modelled population**

We simulated a population of HIV-exposed and unexposed infants born in Côte d’Ivoire (CI), South Africa (SA), and Zimbabwe with country-specific maternal HIV prevalence (4.8%, 30.8%, 16.1%), incidence (0.4, 2.9, 1.5/100PY), and knowledge of HIV status (86%, 89%, 84%) [3–8].

**Model input parameters**

For mothers with unknown HIV status who present to an expanded program upon immunization visit, we modelled a screening cost of $10. This cost represents provider-delivered HIV testing and counseling ($3, based on a $1.47 test kit cost derived from the UNICEF catalogue and the need for additional tests to confirm positive first tests) and program implementation ($7, based on published costing literature) [9–11].

**Model structure**

The Cost-Effectiveness of Preventing AIDS Complications (CEPAC)-Pediatric model is a Monte Carlo simulation model of HIV acquisition, diagnosis, disease progression, and treatment in children. We have previously reported the structure of the CEPAC-Pediatric natural history model, reflecting HIV disease progression in the absence of antiretroviral therapy (ART) [12]. In later manuscripts and corresponding Appendices, we provided additional detail about HIV treatment and early infant diagnosis in the CEPAC-Pediatric model [1,2,13].

Here, we highlight the description of key model features from these prior publications and provide additional detail about early infant diagnosis (EID) in the CEPAC-Pediatric model [1,2].

Key simulated health states include infants not at risk for HIV, infants at risk for HIV (due to maternal HIV during pregnancy and/or breastfeeding), and infants with HIV (HIV acquired *in utero*, during delivery, or during breastfeeding). For infants with HIV, key health states include undiagnosed and diagnosed; once diagnosed, infants can be unlinked to care, linked to care but not on ART, linked to care and on ART, or lost to follow-up (LTFU, assumed to lead to cessation of both care and ART).

Transition probabilities between health states, and clinical outcomes and costs associated with each health state, are modelled as a function of detailed clinical parameters that change over time. Children who are HIV-exposed face a risk of intrauterine or intrapartum HIV acquisition dependent on maternal ART use during pregnancy (reflecting prevention of mother-to-child transmission [PMTCT] coverage) and CD4 count (reflecting disease stage). Children who are HIV-exposed but uninfected face a monthly risk of postnatal HIV acquisition based on maternal ART use, infant antiretroviral prophylaxis, and maternal disease stage (including acute infection during breastfeeding) until cessation of breastfeeding, and no risk thereafter. All simulated children face monthly risks of non-HIV-related mortality. CWH face additional risks of opportunistic infections (OIs), and OI- and HIV-related mortality based on their CD4% (age <5 years) or CD4 count (age ≥5 years), retention in care, and ART use.

*Maternal cohort characteristics and mother-to-child HIV transmission (MTCT)*

The CEPAC-Pediatric model simulates a cohort of infants from birth through death. At the start of each simulation, each infant is assigned a set of maternal characteristics, including maternal HIV status (chronically infected before pregnancy, acutely infected during pregnancy, or not infected; if not infected in pregnancy, probability of acute infection during breastfeeding). Among chronically or acutely infected mothers, we specify the probability of HIV testing and availability of ART during pregnancy and breastfeeding. Maternal HIV disease status (CD4 > or ≤350/µL) and use of ART determine the risk of HIV transmission during three time periods: intrauterine (one-time risk), intrapartum (one-time risk), and postpartum (monthly risk until weaning).

*Untreated pediatric HIV infection*

At the time of infant HIV infection, infants draw from distributions of HIV RNA and CD4 levels; the model uses CD4 percentage (CD4%) for children <5 years old and absolute CD4 count thereafter. Current age and CD4% or CD4 count in each month determine the risks of disease progression, including development of acute OIs and death. Without effective ART, CD4% or CD4 count declines monthly. The model tracks true CD4%/CD4 and HIV RNA level, although clinical decisions are made based on observed information, such as symptomatic illness or CD4%/CD4 or RNA levels (measured according to user-specified laboratory monitoring strategies). In each month, children can remain in care or be lost to follow-up; if they are lost to follow-up, they are assumed to stop ART, and to return to care if a severe OI occurs.

*Treated pediatric HIV infection: ART initiation, efficacy, monitoring, and switching*

The model includes criteria by which children can initiate first-line ART, including age, observed CD4% or CD4 count, and/or development of OIs; in the base-case, all children are treated after diagnosis and linkage to care, regardless of these characteristics. For each ART regimen, we specify an “efficacy,” defined as the probability of suppressing HIV RNA to <400 copies/mL, and the time point by which this occurs (usually 24 or 48 weeks). Each regimen also confers monthly medication costs, as well as gains in CD4% or CD4 count for children with suppressed HIV RNA. Children who initially suppress HIV RNA at 24 or 48 weeks face a monthly risk of virologic failure thereafter (“late failure”). Following virologic failure, HIV RNA slowly rises to a “set point” that is determined as a function of HIV RNA level at birth. After virologic failure, there is a user-specified (base-case: 12-month) delay until CD4% or CD4 count begins to decline at pre-ART rates, leading to increased monthly risks of OIs and death, until the next effective ART regimen (if available) is initiated. For children who fail each modelled line of ART, we assign clinical criteria (number and type of OIs), immunologic criteria (decline in CD4% or CD4 count), or virologic criteria (increase in HIV RNA) by which this failure can be detected, as well as the type and frequency of monitoring and confirmatory testing. After observed failure, patients can be switched to the next available line of therapy. We also incorporate a reduction in mortality and OI risks for children on ART, independent of CD4 level and HIV RNA suppression, as observed in adults; this parameter was used for model calibration [10,11].

Detection and confirmation of first-line ART failure was only possible after more than 24 weeks on ART and was modelled using the criteria below:

- Virologic failure: Observed RNA ≥1,000 copies/mL, confirmed by a second RNA test at least 1 month after the first
- Immunologic failure: Observed CD4% <10% (for children <5 years old) or CD4 count <100/µL (for children ≥5 years old)
- Clinical failure: Observed new or recurring WHO Stage 3/4 or TB event
- When immunologic or clinical failure was observed, the ART regimen was not switched until failure was confirmed with a subsequent virologic test

*PCR sensitivity*

We modelled PCR sensitivity as a function of time since infection to reflect the threshold of viremia necessary for detection: PCR at birth detected intrauterine but not intrapartum infection; PCR at 6 weeks detected intrauterine infection, intrapartum infection, and postpartum infections occurring at least 2 weeks prior.

***Loss to follow-up and return to care***

Infants face a monthly probability of being lost to care, and, subsequently, a monthly probability of returning to care (with potential to return to care following a severe OI). If children are lost to follow-up, they are assumed to stop ART and experience viral rebound.

**Sample Patient’s CD4 over Time**

**CD4% or absolute CD4**

Full details of model structure and data sources, as well as procedures for initiating new collaborative projects are also available on the MPEC website, at https://mpec.massgeneral.org.

**Model validation and calibration**

The CEPAC-Pediatric model has been previously validated, as described in Ciaranello *et al*., *PLoS One* 2013, and calibrated to fit observed survival and opportunistic infection (OI) data for HIV-infected children and adults treated and not treated with ART [12,13,15,16]. To assess model structure and accuracy (i.e., internal validation), we used input data from the International Epidemiologic Database to Evaluate AIDS (IeDEA) East Africa region to verify that model output (e.g., projected survival and OI rates) matched these input data [17].

Given that the IeDEA East African cohort represents a highly selected population of children with excellent access to HIV care, we then calibrated the model to fit observed data for untreated children in other African settings. We calibrated survival rates to 12 PMTCT studies (pooled by the UNAIDS Child Survival Group) representing untreated CWH, infected intrauterine or intrapartum, from eight sub-Saharan African countries [18,19]. To calibrate to these data, we systematically varied HIV-related mortality to represent variations in treatment availability between this cohort and the IeDEA cohort [12].

For CWH treated on ART, we calibrated our model to match mortality, OI rates, and rates of switching from first-line to second-line ART. We used data from the International Maternal, Pediatric, and Adolescent Clinical Trial (P1060) to produce calibrated relative risk reductions of 90% for mortality and 85% for OIs for children (<13 years) on ART (Table S1) [13,14]. These parameters were varied extensively during calibration and were selected on the basis of producing model-generated mortality and OI rates most similar to the P1060 data. A similar process was used to estimate mortality and OI relative risk reductions for adults (>13 years) using data from the Cotrimo-CI ANRS 059 and Cotrame ANRS 1203 studies (Table S1) [14].

To model switching from first-line to second-line ART, we followed the WHO virologic, immunologic, and clinical failure criteria (the most recent criteria are described above). The published literature provides a wide range of reported rates of switching to second-line ART [20–27]. To best model ART-switching strategies that were based on current guidelines but also realistic in field settings, we previously modelled three ART switching scenarios: 1) immediate switch to second-line ART after confirmation of first-line ART failure, 2) deliberate 6-month delay after confirmation of first-line ART failure and initiation of second-line ART, and 3) 6-month delay after confirmation of first-line ART failure (without HIV RNA monitoring) and before initiation of second-line ART. These scenarios are well described in Ciaranello *et al*., *AIDS* 2015. To capture the delay in clinical practice between detecting failure and switching to second-line ART (related to attempts to improve adherence, concerns about toxicity of second-line ART, or lack of available second-line ART), we included a 6-month delay between confirmation of failure and switch to second-line ART in our calibrated model.

**Model outcomes**

For each simulated infant, the model tracks clinical events, changes in CD4% or CD4 count, and the amount of time spent in each health state. After an individual simulated patient has died, the next infant enters the model. Once the entire cohort has been simulated, summary statistics are tallied, including number and type of clinical events, the proportion alive each month, health care costs in each month, and life expectancy (mean for the entire birth cohort). For EID analyses, the model also reports number of HIV-infected infants detected and not detected at key time points, number of each type of EID assay performed, false positive and false negative EID results, and EID assay costs.

**RESULTS**

In the main manuscript, we show the results of the base case analysis, as well as sensitivity analyses in which the results and policy conclusions differed from the base case. All other sensitivity analyses listed in the Appendix Methods above did not lead to changes in policy conclusions (available upon request), except where noted in the manuscript.

**REFERENCES**

1 Francke JA, Penazzato M, Hou T, Abrams EJ, MacLean RL, Myer L, *et al.* Clinical impact and cost-effectiveness of diagnosing HIV infection during early infancy in South Africa: Test timing and frequency. *J Infect Dis* 2016; 214:1319–1328.

2 Dunning L, Francke JA, Mallampati D, MacLean RL, Penazzato M, Hou T, *et al.* The value of confirmatory testing in early infant HIV diagnosis programmes in South Africa: A cost-effectiveness analysis (See Online Appendix, Table S2, for detailed list of ISPOR recommendations and CEPAC approaches to each). *PLoS Med* 2017; 14. doi:10.1371/journal.pmed.1002446.

3 Joint United Nations Programme on HIV/AIDS. UNAIDS data 2018. https://www.aidsdatahub.org/sites/default/files/publication/UNAIDS_Data_2018.pdf

4 South Africa National Department of Health. National antenatal sentinel HIV & syphilis survey report. 2015.

5 Drake AL, Wagner A, Richardson B, John-Stewart G. Incident HIV during pregnancy and postpartum and risk of mother-to-child HIV transmission: a systematic review and meta-analysis. *PLoS Med* 2014; 11:e1001608.

6 World Health Organization. HIV country intelligence: HIV country profiles. 2017.http://cfs.hivci.org/index.html

7 National Department of Health, Statistics South Africa, South African Medical Research Council, The DHS Program, ICF. South Africa demographic and health survey. 2016.https://dhsprogram.com/pubs/pdf/FR337/FR337.pdf

8 Zimbabwe National Statistics Agency, The DHS Program, ICF International. Zimbabwe demographic and health survey. 2015.https://dhsprogram.com/pubs/pdf/FR322/FR322.pdf

9 UNICEF. UNICEF Supply Catalogue. 2018.https://supply.unicef.org/all-materials.html (accessed 23 Oct2018).

10 Bassett IV, Giddy J, Nkera J, Wang B, Losina E, Lu Z, *et al.* Routine voluntary HIV testing in Durban, South Africa: the experience from an outpatient department. *J Acquir Immune Defic Syndr* 2007; 46:181–186.

11 Eaton JW, Menzies NA, Stover J, Cambiano V, Chindelevitch L, Cori A, *et al.* Health benefits, costs, and cost-effectiveness of earlier eligibility for adult antiretroviral therapy and expanded treatment coverage: a combined analysis of 12 mathematical models. *Lancet Glob Health* 2014; 2:e23-34.

12 Ciaranello AL, Morris BL, Walensky RP, Weinstein MC, Ayaya S, Doherty K, *et al.* Validation and calibration of a computer simulation model of pediatric HIV infection. *PLoS ONE* 2013; 8:e83389.

13 Ciaranello AL, Doherty K, Penazzato M, Lindsey JC, Harrison L, Kelly K, *et al.* Cost-effectiveness of first-line antiretroviral therapy for HIV-infected African children less than 3 years of age. *AIDS* 2015; 29:1247–1259.

14 Losina E, Yazdanpanah Y, Deuffic-Burban S, Wang B, Wolf LL, Messou E, *et al.* The independent effect of highly active antiretroviral therapy on severe opportunistic disease incidence and mortality in HIV-infected adults in Côte d’Ivoire. *Antivir Ther (Lond)* 2007; 12:543–551.

15 Ciaranello AL, Lockman S, Freedberg KA, Hughes M, Chu J, Currier J, *et al.* First-line antiretroviral therapy after single-dose nevirapine exposure in South Africa: a cost-effectiveness analysis of the OCTANE trial. *AIDS* 2011; 25:479–492.

16 Walensky RP, Ross EL, Kumarasamy N, Wood R, Noubary F, Paltiel AD, *et al.* Cost-effectiveness of HIV treatment as prevention in serodiscordant couples. *N Engl J Med* 2013; 369:1715–1725.

17 Ciaranello A, Lu Z, Ayaya S, Losina E, Musick B, Vreeman R, *et al.* Incidence of WHO stage 3 and 4 events, tuberculosis, and mortality in untreated, HIV-infected children enrolling in care before 1 year of age: An IeDEA (International epidemiologic Databases to Evaluate AIDS) East Africa regional analysis. *Pediatr Infect Dis J* 2014; 33:623–629.

18 Becquet R, Marston M, Dabis F, Moulton LH, Gray G, Coovadia HM, *et al.* Children who acquire HIV infection perinatally are at higher risk of early death than those acquiring infection through breastmilk: a meta-analysis. *PLoS ONE* 2012; 7:e28510.

19 Marston M, Becquet R, Zaba B, Moulton LH, Gray G, Coovadia H, *et al.* Net survival of perinatally and postnatally HIV-infected children: a pooled analysis of individual data from sub-Saharan Africa. *Int J Epidemiol* 2011; 40:385–396.

20 PENPACT-1 (PENTA 9/PACTG 390) Study Team, Babiker A, Castro nee Green H, Compagnucci A, Fiscus S, Giaquinto C, *et al.* First-line antiretroviral therapy with a protease inhibitor versus non-nucleoside reverse transcriptase inhibitor and switch at higher versus low viral load in HIV-infected children: an open-label, randomised phase 2/3 trial. *Lancet Infect Dis* 2011; 11:273–283.

21 Sauvageot D, Schaefer M, Olson D, Pujades-Rodriguez M, O’Brien DP. Antiretroviral therapy outcomes in resource-limited settings for HIV-infected children <5 years of age. *Pediatrics* 2010; 125:e1039-1047.

22 Judd A, European Pregnancy and Paediatric HIV Cohort Collaboration (EPPICC) study group in EuroCoord. Early antiretroviral therapy in HIV-1-infected infants, 1996-2008: treatment response and duration of first-line regimens. *AIDS (London, England)* 2011; 25:2279–2287.

23 Prendergast AJ, Penazzato M, Cotton M, Musoke P, Mulenga V, Abrams EJ, *et al.* Treatment of young children with HIV infection: using evidence to inform policymakers. *PLoS Med* 2012; 9:e1001273. PMCID: PMC3404108.

24 Walker AS, Doerholt K, Sharland M, Gibb DM, Collaborative HIV Paediatric Study (CHIPS) Steering Committee. Response to highly active antiretroviral therapy varies with age: the UK and Ireland Collaborative HIV Paediatric Study. *AIDS (London, England)* 2004; 18:1915–1924.

25 Davies M-A, Moultrie H, Eley B, Rabie H, Van Cutsem G, Giddy J, *et al.* Virologic failure and second-line antiretroviral therapy in children in South Africa--the IeDEA Southern Africa collaboration. *Journal of Acquired Immune Deficiency Syndromes (1999)* 2011; 56:270–278.

26 Bacha T, Tilahun B, Worku A. Predictors of treatment failure and time to detection and switching in HIV-infected Ethiopian children receiving first line anti-retroviral therapy. *BMC infectious diseases* 2012; 12:197.

27 Cotton MF, Violari A, Otwombe K, Panchia R, Dobbels E, Rabie H, *et al.* Early time-limited antiretroviral therapy versus deferred therapy in South African infants infected with HIV: results from the children with HIV early antiretroviral (CHER) randomised trial. *Lancet (London, England)* 2013; 382:1555–1563.

28 Carter RJ, Dugan K, El-Sadr WM, Myer L, Otieno J, Pungpapong N, *et al.* CD4+ cell count testing more effective than HIV disease clinical staging in identifying pregnant and postpartum women eligible for antiretroviral therapy in resource-limited settings. *JAIDS Journal of Acquired Immune Deficiency Syndromes* 2010; 55:404–410.

29 Ciaranello AL, Myer L, Kelly K, Christensen S, Daskilewicz K, Doherty K, *et al.* Point-of-care CD4 testing to inform selection of antiretroviral medications in South African antenatal clinics: a cost-effectiveness analysis. *PLoS ONE* 2015; 10:e0117751.

30 Kilewo C, Karlsson K, Ngarina M, Massawe A, Lyamuya E, Swai A, *et al.* Prevention of mother-to-child transmission of HIV-1 through breastfeeding by treating mothers with triple antiretroviral therapy in Dar es Salaam, Tanzania: the Mitra Plus study. *J Acquir Immune Defic Syndr* 2009; 52:406–416.

31 Shapiro RL, Hughes MD, Ogwu A, Kitch D, Lockman S, Moffat C, *et al.* Antiretroviral regimens in pregnancy and breast-feeding in Botswana. *N Engl J Med* 2010; 362:2282–2294.

32 Kesho Bora Study Group, de Vincenzi I. Triple antiretroviral compared with zidovudine and single-dose nevirapine prophylaxis during pregnancy and breastfeeding for prevention of mother-to-child transmission of HIV-1 (Kesho Bora study): a randomised controlled trial. *Lancet Infect Dis* 2011; 11:171–180.

33 Tonwe-Gold B, Ekouevi DK, Viho I, Amani-Bosse C, Toure S, Coffie PA, *et al.* Antiretroviral treatment and prevention of peripartum and postnatal HIV transmission in West Africa: evaluation of a two-tiered approach. *PLoS Med* 2007; 4:e257.

34 Fawzi W, Msamanga G, Spiegelman D, Renjifo B, Bang H, Kapiga S, *et al.* Transmission of HIV-1 through breastfeeding among women in Dar es Salaam, Tanzania. *J Acquir Immune Defic Syndr* 2002; 31:331–338.

35 Petra Study Team. Efficacy of three short-course regimens of zidovudine and lamivudine in preventing early and late transmission of HIV-1 from mother to child in Tanzania, South Africa, and Uganda (Petra study): a randomised, double-blind, placebo-controlled trial. *Lancet* 2002; 359:1178–1186.

36 Leroy V, Karon JM, Alioum A, Ekpini ER, Meda N, Greenberg AE, *et al.* Twenty-four month efficacy of a maternal short-course zidovudine regimen to prevent mother-to-child transmission of HIV-1 in West Africa. *AIDS* 2002; 16:631–641.

37 Chigwedere P, Seage GR, Lee T-H, Essex M. Efficacy of antiretroviral drugs in reducing mother-to-child transmission of HIV in Africa: a meta-analysis of published clinical trials. *AIDS Res Hum Retroviruses* 2008; 24:827–837.

38 Dabis F, Bequet L, Ekouevi DK, Viho I, Rouet F, Horo A, *et al.* Field efficacy of zidovudine, lamivudine and single-dose nevirapine to prevent peripartum HIV transmission. *AIDS* 2005; 19:309–318.

39 Scott GB, Brogly SB, Muenz D, Stek AM, Read JS, International Maternal Pediatric Adolescent AIDS Clinical Trials Group (IMPAACT) P1025 Study Team. Missed opportunities for prevention of mother-to-child transmission of human immunodeficiency virus. *Obstet Gynecol* 2017; 129:621–628.

40 Thior I, Lockman S, Smeaton LM, Shapiro RL, Wester C, Heymann SJ, *et al.* Breastfeeding plus infant zidovudine prophylaxis for 6 months vs formula feeding plus infant zidovudine for 1 month to reduce mother-to-child HIV transmission in Botswana: a randomized trial: the Mashi Study. *JAMA* 2006; 296:794–805.

41 Peltier CA, Ndayisaba GF, Lepage P, van Griensven J, Leroy V, Pharm CO, *et al.* Breastfeeding with maternal antiretroviral therapy or formula feeding to prevent HIV postnatal mother-to-child transmission in Rwanda. *AIDS* 2009; 23:2415–2423.

42 Palombi L, Marazzi MC, Voetberg A, Magid NA. Treatment acceleration program and the experience of the DREAM program in prevention of mother-to-child transmission of HIV. *AIDS* 2007; 21 Suppl 4:S65-71.

43 Thomas TK, Masaba R, Borkowf CB, Ndivo R, Zeh C, Misore A, *et al.* Triple-antiretroviral prophylaxis to prevent mother-to-child HIV transmission through breastfeeding--the Kisumu Breastfeeding Study, Kenya: a clinical trial. *PLoS Med* 2011; 8:e1001015.

44 Chasela CS, Hudgens MG, Jamieson DJ, Kayira D, Hosseinipour MC, Kourtis AP, *et al.* Maternal or infant antiretroviral drugs to reduce HIV-1 transmission. *N Engl J Med* 2010; 362:2271–2281.

45 Iliff PJ, Piwoz EG, Tavengwa NV, Zunguza CD, Marinda ET, Nathoo KJ, *et al.* Early exclusive breastfeeding reduces the risk of postnatal HIV-1 transmission and increases HIV-free survival. *AIDS* 2005; 19:699–708.

46 Kuhn L, Aldrovandi GM, Sinkala M, Kankasa C, Semrau K, Mwiya M, *et al.* Effects of early, abrupt weaning on HIV-free survival of children in Zambia. *N Engl J Med* 2008; 359:130–141.

47 Kuhn L, Aldrovandi GM, Sinkala M, Kankasa C, Mwiya M, Thea DM. Potential impact of new WHO criteria for antiretroviral treatment for prevention of mother-to- child HIV transmission. *AIDS* 2010; 24:1374–1377.

48 Mallampati D, Ford N, Hannaford A, Sugandhi N, Penazzato M. Performance of virological testing for early infant diagnosis: a systematic review. *J Acquir Immune Defic Syndr* 2017; 75:308–314.

49 World Health Organization. New strategies for infant HIV diagnosis: expert review meeting. 2013.

50 Hsiao N, Dunning L, Kroon M, Myer L. Laboratory evaluation of the Alere q point-of-care system for early infant HIV diagnosis. *PLoS ONE* 2016; 11:e0152672.

51 Jani IV, Meggi B, Mabunda N, Vubil A, Sitoe NE, Tobaiwa O, *et al.* Accurate early infant HIV diagnosis in primary health clinics using a point-of-care nucleic acid test. *J Acquir Immune Defic Syndr* 2014; 67:e1-4.

52 World Health Organization. WHO prequalification of diagnostics programme public report; product: Alere Determine^TM^ HIV-1/2. ; 2018. https://www.who.int/diagnostics_laboratory/evaluations/pq-list/hiv-rdts/180913_amended_final_pqpr_0033_013_00_v6.pdf?ua=1 (accessed 23 Oct2019).

53 Faraoni S, Rocchetti A, Gotta F, Ruggiero T, Orofino G, Bonora S, *et al.* Evaluation of a rapid antigen and antibody combination test in acute HIV infection. *J Clin Virol* 2013; 57:84–87.

54 Violari A, Lindsey JC, Hughes MD, Mujuru HA, Barlow-Mosha L, Kamthunzi P, *et al.* Nevirapine versus ritonavir-boosted lopinavir for HIV-infected children. *N Engl J Med* 2012; 366:2380–2389.

55 Palumbo P, Lindsey JC, Hughes MD, Cotton MF, Bobat R, Meyers T, *et al.* Antiretroviral treatment for children with peripartum nevirapine exposure. *N Engl J Med* 2010; 363:1510–1520.

56 Ciaranello AL, Chang Y, Margulis AV, Bernstein A, Bassett IV, Losina E, *et al.* Effectiveness of pediatric antiretroviral therapy in resource-limited settings: a systematic review and meta-analysis. *Clin Infect Dis* 2009; 49:1915–1927.

57 Sutcliffe CG, van Dijk JH, Bolton C, Persaud D, Moss WJ. Effectiveness of antiretroviral therapy among HIV-infected children in sub-Saharan Africa. *Lancet Infect Dis* 2008; 8:477–489.

58 Joint United Nations Programme on HIV/AIDS. Progress report on the global plan towards the elimination of new HIV infections among children and keeping their mothers alive. 2015.https://www.unaids.org/sites/default/files/media_asset/JC2774_2015ProgressReport_GlobalPlan_en.pdf

59 Lilian RR, Johnson LF, Moolla H, Sherman GG. A mathematical model evaluating the timing of early diagnostic testing in HIV-exposed infants in South Africa. *J Acquir Immune Defic Syndr* 2014; 67:341–348.

60 Hsiao N-Y, Stinson K, Myer L. Linkage of HIV-infected infants from diagnosis to antiretroviral therapy services across the Western Cape, South Africa. *PLoS ONE* 2013; 8:e55308.

61 McCollum ED, Preidis GA, Kabue MM, Singogo EBM, Mwansambo C, Kazembe PN, *et al.* Task shifting routine inpatient pediatric HIV testing improves program outcomes in urban Malawi: a retrospective observational study. *PLoS ONE* 2010; 5:e9626.

62 World Health Organization, UNICEF. WHO-UNICEF estimates of DTP1 coverage. 2018.https://apps.who.int/immunization_monitoring/globalsummary/timeseries/tswucoveragedtp1.html

63 Rollins N, Mzolo S, Moodley T, Esterhuizen T, van Rooyen H. Universal HIV testing of infants at immunization clinics: an acceptable and feasible approach for early infant diagnosis in high HIV prevalence settings: *AIDS* 2009; 23:1851–1857.

64 Stover J, Glaubius R, Mofenson L, Dugdale CM, Davies M-A, Patten G, *et al.* Updates to the Spectrum/AIM model for estimating key HIV indicators at national and subnational levels: *AIDS* 2019; 33:S227–S234.

65 Mabugu T. Zimbabwe national AIDS spending assessment: consolidated report 2011 and 2012. UNAIDS; 2012.

66 Menzies NA, Berruti AA, Berzon R, Filler S, Ferris R, Ellerbrock TV, *et al.* The cost of providing comprehensive HIV treatment in PEPFAR-supported programs. *AIDS* 2011; 25:1753–1760.

67 Cleary S, Chitha W, Jikwana S, Okorafor O, Boulle A. South African health review 2005. 2005.http://www.hst.org.za/publications/South%20African%20Health%20Reviews/sahr05.pdf. (accessed 21 Dec2018).

68 Thomas LS. Costing of HIV/AIDS services at a tertiary level hospital in Gauteng Province. *Faculty of Health Sciences, University of Witwatersrand, South Africa* Published Online First: 2006.http://wiredspace.wits.ac.za/bitstream/handle/10539/2008/Dr%20L%20S%20Thomas%20MMed%20Report.pdf?sequence=2&isAllowed=y. (accessed 21 Dec2018).

69 Desmonde S, Avit D, Petit J, Amorissani Folquet M, Eboua FT, Amani Bosse C, *et al.* Costs of care of HIV-infected children initiating lopinavir/ritonavir-based antiretroviral therapy before the age of two in Côte d’Ivoire. *PLoS ONE* 2016; 11. doi:10.1371/journal.pone.0166466

70 Clinton Health Access Initiative. 2017 antiretroviral (ARV) CHAI reference price list. Clinton Health Access Initiative. 2017.https://clintonhealthaccess.org/2017-chai-arv-reference-price-list/. (accessed 21 Dec2018).

71 Doherty K, Essajee S, Penazzato M, Holmes C, Resch S, Ciaranello A. Estimating age-based antiretroviral therapy costs for HIV-infected children in resource-limited settings based on World Health Organization weight-based dosing recommendations. *BMC Health Serv Res* 2014; 14:201.

72 The Global Fund. HIV viral load and early infant diagnosis selection and procurement information tool. 2017.https://www.theglobalfund.org/media/5765/psm_viralloadearlyinfantdiagnosis_content_en.pdf

73 Clinton Health Access Initiative. HIV/AIDS diagnostic pricing outlook. ; 2009.

74 Ouattara EN, MacLean RL, Danel C, Borre ED, Gabillard D, Huang M, *et al.* Cost-effectiveness and budget impact of immediate antiretroviral therapy initiation for treatment of HIV infection in Côte d’Ivoire: A model-based analysis. *PLoS ONE* 2019; 14:e0219068.

75 Cambiano V, Ford D, Mabugu T, Napierala Mavedzenge S, Miners A, Mugurungi O, *et al.* Assessment of the potential impact and cost-effectiveness of self-testing for HIV in low-income countries. *J Infect Dis* 2015; 212:570–577.

76 The World Bank. Data: Birth rate, crude (per 1,000 people). 2017.https://data.worldbank.org/indicator/SP.DYN.CBRT.IN (accessed 1 Oct2020).

77 Molebatsi T, Lehloenya G. Statistics South Africa. Recorded live births, 2017. 2018.http://www.statssa.gov.za/?p=11478&gclid=EAIaIQobChMIu8H5i_Gb7AIVgfGzCh25IAV6EAAYASAAEgLnXPD_BwE (accessed 1 Oct2020).

| **Variable** | **Base case value [range examined] ^†^** | | **References** |
| --- | --- | --- | --- |
| **I. CLINICAL INPUT PARAMETERS** |  | |  |
| Age, months (SD) | 0 (0) | | Assumption |
| Male infants, % | 48 | | [17] |
| Mothers with CD4 ≤350/µL before ART, % | 49 | | [28] |
| Infant CD4% at infection, mean (SD) | 45 (10) | | [12] |
| Monthly maternal mortality probability, % | 0.1 | | [29] |
| IU/IP MTCT (one-time risk in pregnancy/delivery, %) | Maternal CD4 ≤350 | Maternal CD4 >350 |  |
| On ART | 0.93 | 0.93 | [30–33] |
| Not on ART | 27 | 17 | [34–39] |
| PP MTCT (monthly risk during breastfeeding, %) |  | |  |
| On ART | 0.19 | 0.19 | [40–44] |
| Not on ART |  | |  |
| Exclusive breastfeeding | 0.76 | 0.24 | [34,45–47] |
| Mixed or complementary breastfeeding | 1.28 | 0.40 | [34,45–47] |
| **II. ASSAY CHARACTERISTICS** |  |  |  |
| NAT sensitivity, specificity for infant HIV, % |  |  |  |
| IU infection: all ages, % | 100, 99.6 | | [48–51] |
| IP/PP infection: month in which infection occurs, % | 0, 99.6 | | [48–51] |
| IP/PP infection: subsequent months, % | 100, 99.6 | | [48–51] |
| RDT sensitivity, specificity for maternal HIV, % | 99.9, 100 | | [52,53] |

**TABLE S1: Base case data parameters and ranges for the CEPAC-Pediatric model analysis of *EID* and *screen-and-test***

**TABLE S1: Base case data parameters and ranges for the CEPAC-Pediatric model analysis of *EID* and *screen-and-test* (continued)**

| **Variable** | **Base case value [range examined] ^†^** | | | | **References** |
| --- | --- | --- | --- | --- | --- |
| **III. ART OUTCOMES** | (1^st^-line ART) | | (2^nd^-line ART) | |  |
| ART efficacy: HIV RNA <400c/mL at 24 weeks on ART, % |  | |  | |  |
| Ages <5 years | 91 | | 75 | | [54,55] |
| Ages ≥5 years | 75 | | 75 | | [20] |
| ART-associated CD4-independent risk reductions, % |  | |  | |  |
| Risk reduction in opportunistic infection (ages <13 years) | 85  32  90  55-96 | | | | [13] |
| Risk reduction in opportunistic infection (ages ≥13 years) |  |  |  |  | [14] |
| Risk reduction in mortality (ages <13 years) |  |  |  |  | [13] |
| Risk reduction in mortality (ages ≥13 years, range by CD4) |  |  |  |  | [14] |
| Monthly loss to follow-up after ART initiation, % | 0.2 | | | | [56,57] |
| **IV. COUNTRY-SPECIFIC CLINICAL PARAMETERS** | **Côte d’Ivoire** | **South Africa** | | **Zimbabwe** |  |
| Antenatal: |  |  | |  |  |
| Maternal HIV prevalence, % | 4.8 [2-9] | 30.8 [19-44] | | 16.1 [14-18] | [3,4] |
| Maternal knowledge of HIV status, % ^‡^ | 86 [42-86] | 89 [72-89] | | 84 [69-85] | [3,6–8] |
| Postnatal: |  |  | |  |  |
| Maternal HIV incidence (/100PY) | 0.4 [0.2-0.8] | 2.9 [1.8-4.0] | | 1.5 [1.3-1.6] | [5] |
| Mean breastfeeding duration, months | 12 [0-36] | 12 [0-36] | | 18 [0-36] | [7,8,58] |

**Table S1: Select base case data parameters and ranges for the CEPAC-Pediatric model analysis of *EID* and *screen-and-test* (continued)**

| **Variable** | **Base case value [range examined] ^†^** | | | **References** |
| --- | --- | --- | --- | --- |
| **IV. COUNTRY-SPECIFIC CLINICAL PARAMETERS (cont’d)** | **Côte d’Ivoire** | **South Africa** | **Zimbabwe** |  |
| Postnatal: |  |  |  |  |
| Proportion of infants breastfed from birth (%) | 80 | 80 | 80 | [58,59] |
| Exclusively breastfeeding for 1^st^ 6 months, % | 25 | 55 | 55 | [59] |
| Mixed breastfeeding for 1^st^ 6 months, % | 55 | 25 | 25 | [59] |
| Maternal ART coverage in pregnancy/breastfeeding (PMTCT), % | 70 [45-95] | 95 [82-95] | 95 [77-95] | [3] |
| Infant linkage to care/ART after detection by OI, % | 71 [0-100] | 71 [0-100] | 71 [0-100] | [60,61] |
| Routine 6-week EID for infants with known HIV exposure: |  |  |  |  |
| Uptake of existing EID programs, % | 40 [27-62] | 95 [85-95] | 65 [57-81] | [3] |
| Linkage to care/ART after positive EID test, % | 71 [0-100] | 71 [0-100] | 71 [0-100] | [60,61] |
| Result-return time for confirmed HIV diagnosis, months (SD) | 1 (0) | 1 (0) | 1 (0) | [60] |
| Maternal HIV testing at infant immunization visits: |  |  |  |  |
| Immunization coverage (6-10 weeks), % | 99 [84-99] | 74 [66-74] | 94 [89-94] | [62] |
| Offer and acceptance of maternal RDT, % | 90 | 90 | 90 | [63] |
| Linkage to care/ART for newly diagnosed mothers, % | 80 [30-100] | 80 [57-100] | 80 [77-100] | [3,64] |
| Linkage to NAT for HIV-exposed infants, % | 80 [0-100] | 80 [0-100] | 80 [0-100] | Assumption |
| Linkage to care/ART for diagnosed infants referred from EPI, % | 71 [0-100] | 71 [0-100] | 71 [0-100] | [60,61] |

**Table S1: Select base case data parameters and ranges for the CEPAC-Pediatric model analysis of *EID* and *screen-and-test***

**(continued)**

| **Variable** | **Base case value [range examined] ^†^** | | | **References** |
| --- | --- | --- | --- | --- |
| **V. COSTS (2018 USD)** | **Côte d’Ivoire** | **South Africa** | **Zimbabwe** |  |
| Routine HIV care, per month (range by CD4%/CD4) | 20-190  [0.5-2x] | 55-140  [0.5-2x] | 30-35  [0.5-2x] | [65–69] |
| Acute OI care (range by type of OI) | 60-480  [0.5-2x] | 210-1,490 [0.5-2x] | -- ^§^ |  |
| Pediatric ART, per month (range by ART regimen) | 5-31  [0.5-2x] | 5-31  [0.5-2x] | 5-31  [0.5-2x] | [70,71] |
| NAT, per assay | 24  [12.50-40] | 24  [12.50-40] | 24  [12.50-40] | [72] |
| CD4 test, per assay | 10 | 12 | 5 | [67,73,74] |
| HIV RNA test, per assay | 32 | 23 | 17 | [67,72,74] |
| Maternal screening program, per mother-infant pair ^¶^ | 10 [5-60] | 10 [5-60] | 10 [5-60] | [75] |

^†^ Ranges examined reflect plausible variations in parameters.

^‡^ Maternal knowledge of HIV status was calculated from the product of ANC coverage and frequency of HIV testing in ANC in each country.

^§^ Based on available data, for CI and SA we modelled costs of care for individual OIs; in Zimbabwe, OI care was included in overall monthly care costs.

^¶^ Overall cost reflects both the cost of a maternal rapid diagnostic test and program implementation costs.

Abbreviations: ANC, antenatal coverage; ART, antiretroviral therapy; EID, early infant diagnosis; EPI, expanded program on immunization; IP, intrapartum; IU, intrauterine; MTCT, mother-to-child transmission; NAT, nucleic acid test; OI, opportunistic infection; PP, post-partum; PY, person-years; RDT, rapid diagnostic test; SD, standard deviation.

**TABLE S2: Discounted life expectancy for the birth cohort in Côte d’Ivoire, South Africa, and Zimbabwe**

|  | Life expectancy per person (discounted years) | Recorded births in 2017  (number) | Life-years saved in birth cohort (discounted years) ^§^ |
| --- | --- | --- | --- |
| **CÔTE D’IVOIRE** |  |  |  |
| *EID* | 26.86 | 879,577 ^†^ | 17,590 |
| *Screen-and-test* | 26.87 |  |  |
| **SOUTH AFRICA** |  |  |  |
| *EID* | 26.51 | 989,318 ^‡^ | 29,680 |
| *Screen-and-test* | 26.54 |  |  |
| **ZIMBABWE** |  |  |  |
| *EID* | 26.42 | 451,760 ^†^ | 13,550 |
| *Screen-and-test* | 26.45 |  |  |

^†^ The recorded number of births in Côte d’Ivoire and Zimbabwe in 2017 was obtained from the World Bank [76].

^‡^ The recorded number of births in South Africa in 2017 was obtained from Statistics South Africa [77].

^§^ Rounded to the nearest 10 years.

**TABLE S3: Outcomes and mechanisms of HIV detection among children ever infected with HIV at 1 year from birth in the *screen-and-test* strategy in Côte d’Ivoire, South Africa, and Zimbabwe**

| **Outcome** | **Côte d’Ivoire** | **South Africa** | **Zimbabwe** |
| --- | --- | --- | --- |
| **Dead, % of all infants ever infected** | **28** | **30** | **29** |
| **Alive and infected IU/IP, % of all infants ever infected** | **52** | **44** | **46** |
| Undetected, % | 14 | 17 | 14 |
| Detected by existing EID programs, % | 53 | 43 | 37 |
| Detected by *screen-and-test*, % | 16 | 21 | 32 |
| Detected upon presentation with an OI, % | 17 | 19 | 17 |
| **Alive and infected PP, % of all infants ever infected** | **20** | **26** | **24** |
| Undetected, % | 76 | 77 | 78 |
| Detected by existing EID programs, % | 10 | 9 | 9 |
| Detected by *screen-and-test*, % | 1 | 1 | 1 |
| Detected upon presentation with an OI, % | 13 | 13 | 12 |

Abbreviations: EID, early infant detection; IP, intrapartum; IU, intrauterine; OI, opportunistic infection; PP, postpartum.

**FIGURE LEGENDS**

**FIGURE S1. Two-year survival of infants with HIV diagnosed by *EID* only and with addition of *screen-and-test* in Côte d’Ivoire (top left panel), South Africa (top right panel), and Zimbabwe (bottom left panel)**

One-year survival among infants diagnosed with HIV by *EID* only (blue) and *screen-and-test* (grey). Survival is not expected to be directly comparable across country settings.

**FIGURE S2. Total lifetime costs per infant by HIV testing strategy in Côte d’Ivoire, South Africa, and Zimbabwe**

Bar graphs representing the breakdown of lifetime costs, per infant of the complete birth cohort, in *EID* and *screen-and-test*. In *screen-and-test*, the screening program (at $10/mother-infant pair) comprised 13% (Côte d’Ivoire), 6% (South Africa), and 9% (Zimbabwe) of all lifetime costs per infant. Of the *additional* costs associated with *screen-and-test* compared to *EID*, 42% (Côte d’Ivoire), 33% (South Africa), and 42% (Zimbabwe) was due to testing, and the remaining was due to HIV care and ART. When limited to only children with HIV (not shown), the screening program accounted for 1% of the additional cost of *screen-and-test* compared to *EID*; 99% of the cost increase was due to HIV care and ART. All costs are undiscounted and reported in 2018 USD. Abbreviations: ART, antiretroviral therapy; EID, early infant detection; NAT, nucleic acid test; USD, United States dollar.

**Detailed sources for Table 1, Part IV:**

| **Parameter** | **Côte d’Ivoire** | **Data Source/Derivation** |
| --- | --- | --- |
| Maternal prevalence | 4.8% | Drake 2014, UNAIDS 2018  (ratio of maternal prevalence in SA to prevalence among women 15-49 in SA) * (prevalence among women 15-49 in CI) |
| Maternal incidence post-partum (/year) | 0.4% | Drake 2014  (ratio of maternal incidence in SA to maternal prevalence in SA) * CDI prevalence |
| Maternal knowledge of HIV status during pregnancy | 86% | WHO country profile 2017, UNAIDS 2018  ANC coverage (91%) * Maternal HIV testing (95%) |
| Maternal ART coverage during pregnancy if known to be HIV-infected | 70% | UNAIDS 2018 |
| Mean breastfeeding duration, months | 12m | Assumption |
| Uptake of existing EID programs for infants with known HIV-exposure at 6 weeks | 40% | UNAIDS 2018 |
| Immunization coverage (6w-10w) | 99% | WHO-UNICEF 2018 estimate of DTP1 (diphtheria, tetanus, and pertussis) coverage |
| Linkage to HIV care for infants found to be HIV-infected at routine EID | 71% | Hsiao 2013 |
| Linkage to NAT for infants found to be HIV-exposed by *screen-and-test* | 80% | Assumption |
| Linkage to HIV care for infants found to be HIV-infected after *screen-and-test* | 71% | Hsiao 2013 |
| ART coverage during breastfeeding if mother’s status becomes known | 56% | Stover 2019, UNAIDS 2018  80% * PMTCT coverage |
| Breastfeeding (proportion of all mother-infant pairs) | 80% | 2015 UNAIDS progress report |
| Exclusively breastfeeding for 1^st^ 6 months | 25% | Lilian 2014, 2015 UNAIDS progress report |
| Mixed breastfeeding for 1^st^ 6 months | 55% | Lilian 2014, 2015 UNAIDS progress report |

**Detailed sources for Table 1, Part IV:**

| **Parameter** | **South Africa** | **Data Source/Derivation** |
| --- | --- | --- |
| Maternal prevalence | 30.8% | National Antenatal Sentinel HIV & Syphilis Survey Report (SA National Department of Health 2015) |
| Maternal incidence post-partum (/year) | 2.9% | Drake 2014 |
| Maternal knowledge of HIV status during pregnancy | 89% | WHO HIV country profile 2017 |
| Maternal ART coverage during pregnancy if known to be HIV-infected | 95% | UNAIDS 2018 (PMTCT) |
| Mean breastfeeding duration, months | 12m | 2015 UNAIDS progress report |
| Uptake of existing EID programs for infants with known HIV-exposure at 6 weeks | 95% | UNAIDS 2018 |
| Immunization coverage (6w-10w) | 74% | WHO-UNICEF 2018 estimate of DTP1 (diphtheria, tetanus, and pertussis) coverage |
| Linkage to HIV care for infants found to be HIV-infected at routine EID | 71% | Hsiao 2013 |
| Linkage to NAT for infants found to be HIV-exposed by *screen-and-test* | 80% | Assumption |
| Linkage to HIV care for infants found to be HIV-infected after *screen-and-test* | 71% | Hsiao 2013 |
| ART coverage during breastfeeding if mother’s status becomes known | 76% | Stover 2019, UNAIDS 2018  80% * PMTCT coverage |
| Breastfeeding (proportion of all mother-infant pairs) | 80% | 2015 UNAIDS progress report |
| Exclusively breastfeeding for 1^st^ 6 months | 55% | Lilian 2014 |
| Mixed breastfeeding for 1^st^ 6 months | 25% | Lilian 2014 |

**Detailed sources for Table 1, Part IV, continued**

| **Parameter** | **Zimbabwe** | **Data Source/Derivation** |
| --- | --- | --- |
| Maternal prevalence | 16.1% | Drake 2014, UNAIDS 2018  (ratio of maternal prevalence in SA to prevalence among women 15-49 in SA) * (prevalence among women 15-49 in CI) |
| Maternal incidence post-partum (/year) | 1.5% | Drake 2014  (ratio of maternal incidence in SA to maternal prevalence in SA) * CDI prevalence |
| Maternal knowledge of HIV status during pregnancy | 84% | WHO country profile 2017, Zimbabwe DHS 2015  ANC coverage (93%) * Maternal HIV testing (90%) |
| Maternal ART coverage during pregnancy if known to be HIV-infected | 95% | UNAIDS 2018 (PMTCT) |
| Mean breastfeeding duration, months | 18m | 2015 UNAIDS progress report |
| Uptake of existing EID programs for infants with known HIV-exposure at 6 weeks | 65% | UNAIDS 2018 |
| Immunization coverage (6w-10w) | 94% | WHO-UNICEF 2018 estimate of DTP1 (diphtheria, tetanus, and pertussis) coverage |
| Linkage to HIV care for infants found to be HIV-infected at routine EID | 71% | Hsiao 2013 |
| Linkage to NAT for infants found to be HIV-exposed | 80% | Assumption |
| Linkage to HIV care for infants found to be HIV-infected after *screen-and-test* | 71% | Hsiao 2013 |
| ART coverage during breastfeeding if mother’s status becomes known | 76% | Stover 2019, UNAIDS 2018  80% * PMTCT coverage |
| Breastfeeding (proportion of all mother-infant pairs) | 80% | 2015 UNAIDS progress report |
| Exclusively breastfeeding for 1^st^ 6 months | 55% | Lilian 2014 |
| Mixed breastfeeding for 1^st^ 6 months | 25% | Lilian 2014 |
